# Robust pleiotropy-decomposed polygenic scores identify distinct contributions to elevated coronary artery disease polygenic risk

**DOI:** 10.1371/journal.pcbi.1013191

**Published:** 2025-06-26

**Authors:** Jiaqi Hu, Yixuan Ye, Chi Zhang, Yunfeng Ruan, Pradeep Natarajan, Hongyu Zhao

**Affiliations:** 1 Department of Chronic Disease Epidemiology, Yale School of Public Health, New Haven, Connecticut, United States of America; 2 Program of Computational Biology and Bioinformatics, Yale University, New Haven, Connecticut, United States of America; 3 Department of Biostatistics, Yale School of Public Health, New Haven, Connecticut, United States of America; 4 Cardiovascular Disease Initiative, Broad Institute of MIT and Harvard, Cambridge, Massachusetts, United States of America; 5 Center for Genomic Medicine, Massachusetts General Hospital, Boston, Massachusetts, United States of America; 6 Harvard Medical School, Boston, Massachusetts, United States of America; 7 Cardiovascular Research Center, Massachusetts General Hospital, Boston, Massachusetts, United States of America; University of Waterloo, CANADA

## Abstract

**Background:**

Polygenic risk score (PRS) have proved to offer robust risk prediction for coronary artery disease (CAD). However, the global CAD PRS summarizes the joint effects of all the markers in the genome, masking potential genetic heterogeneity that may be important for disease interpretation and targeted interventions.

**Methods:**

Using summary-level data, we identified 43 significant CAD-related traits based on genetic correlations, and further classified them into eight pleiotropy clusters based on their biological functions. We then partitioned the genome into 2,353 near-independent regions. Variants in each region were assigned to the trait most genetically similar to CAD, and then were labeled with the corresponding pleiotropy cluster. We grouped variants without labels into a ninth, non-specific cluster. The Pleiotropy Decomposed (PD) PRSs for each of the nine clusters were calculated using variants assigned to each cluster for 407,903 samples of European ancestry from the UK Biobank (UKBB).

**Results:**

We decomposed the CAD PRS into nine PD-PRSs and further stratified individuals with high CAD-PRS into nine subgroups. Each PD-PRS accounted for a higher proportion of the global CAD-PRS within its corresponding subgroup than in the remaining subjects with high CAD-PRS (e.g., 25.2% (0.07) vs. 10.06% (0.07) for lipids-PD-PRS). Additionally, these subgroups showed distinct clinical features. For example, in the lipids-related subgroup, lipoprotein(a) and LDL-cholesterol levels were 67.5% and 18.3% higher, respectively, compared to the remaining high-risk individuals. Furthermore, significant interactions were observed between blood pressure and BP PD-PRS, and between current smoking and respiratory system PD-PRS.

**Conclusion:**

Our findings suggest that PD-PRSs may reveal substantial genetic and phenotypic heterogeneity among individuals with high CAD-PRS. The unique PD-PRS compositions of each individual can highlight the relative importance of different pleiotropic regions.

## Author summary

Aggregating genetic variants into a polygenic risk score (PRS) can predict an individual’s risk for complex diseases such as coronary artery disease (CAD). However, people with high PRS for CAD show different symptoms and clinical outcomes. Understanding disease heterogeneity can help us better understand the genetic etiology of CAD and improve prevention and treatment. The existing methods to decompose CAD PRS into pathway-specific PRSs mainly consider a limited number of SNPs, limiting the overall utility of decompositions. In this study, we propose a framework to assign genome-wide genetic variants to eight relevant clusters and one non-specific cluster based on pleiotropy, where one variant is associated with two or more traits/disease. As a result, we decompose the overall CAD PRS into nine pleiotropy-decomposed PRSs (PD-PRSs) and use them to classify individuals with high CAD PRS into nine subgroups, which show both genetic and phenotypic heterogeneity among high-risk individuals. Additionally, interactions between current smoking and respiratory system-related subgroup indicate potential application of our PD-PRSs in offering clinical advice.

## Introduction

Prevention and early diagnosis are critical to relieving the significant burden of coronary artery disease (CAD), which is a major cause of morbidity and mortality worldwide [1]. As the risk-based prevention strategy is now widely accepted as an efficient way of disease prevention, it is essential to have accurate prediction models for CAD [2]. The past two decades have witnessed significant advances in genome-wide association studies (GWASs), identifying hundreds of single nucleotide polymorphisms (SNPs) associated with CAD [3–6], facilitating the use of genetic markers to predict CAD risk. More recently, polygenic risk scores (PRSs), which quantify individual risk for disease by aggregating estimated effects from multiple SNPs, have been built for CAD, and these PRSs can robustly stratify populations into different risk groups [7]. However, current PRS approaches, which amalgamate effects from all the markers in the human genome, do not distinguish between different trait clusters associated with CAD [8]. Thus, the PRS has been interpreted as an overall risk score for CAD. Identifying these distinct groups among individuals with high CAD risk could reveal heterogeneity among these people and quantify pleiotropy-specific genetic risk, offering better understanding of PRS components and insights for individualized prevention and treatment strategies [9].

In recent years, several studies have sought to decompose a single aggregated PRS into multiple function‐specific component scores, each capturing a distinct pathogenetic mechanism [9–12]. This approach aims to more clearly delineate genetic liabilities arising from different biological pathways, thereby providing finer resolution of how overall genetic risk is distributed across multiple disease‐relevant processes. However, these methods are based on pre-defined pathways, with limited variants linked to genes with known biological functions retained in the decomposed PRSs [11, 12]. Hence there is usually a substantial loss of explained heritability in these PRS decompositions. Alternatively, since pleiotropy may be regarded as the result of some perturbations of the underlying pathways, some researchers have tried to leverage pleiotropy to construct pseudo-pathways, and this strategy was more flexible in investigating potential PRS components [9]. However, these pleiotropy-based analyses are mainly built upon singular value decomposition (SVD) of a matrix with the SNP-phenotype effects, which restricts the number of SNPs that can be included for computation efficiency. Besides, the decomposition is mainly conducted upon the Pruning and Thresholding (P + T) PRS for these analyses, which is significantly less predictive compared to the state-of-the-art PRSs developed from more sophisticated Bayesian frameworks [13–15].

In this study, we propose a novel framework to partition the state-of-the-art CAD PRS into pleiotropy-decomposed PRSs (PD-PRSs) by leveraging local genetic covariances. With the PD-PRSs derived from more than 400,000 UK Biobank (UKBB) individuals of European ancestry, we first investigated the associations between PD-PRSs and a broad range of phenotypes to reveal the biological functions represented by each PD-PRS. We then used the PD-PRSs to explore potential subgroups among individuals with high genetic risk of CAD, and to compare the characteristics of different subgroups. We further investigated the interactions between PD-PRSs and traits of interest to explore whether certain PD-PRS can explain the interaction between traits and the overall PRS. Finally, the interactions were extended to subgroups to further explore the genetic heterogeneity.

## Materials and methods

### Ethics statement

The UK Biobank study had ethical approval from the United Kingdom National Health Service (NHS) Research Ethics Committee (Reference: 11/NW/0382). Signed and informed consent was obtained from all of the participants.

### Study subjects

The UKBB is a large-scale prospective cohort study designed for analyses of both genetic and non-genetic risk factors for diseases among middle-aged to elderly populations [16]. From 2006 to 2010, 502,618 subjects aged 40–69 were recruited from 22 assessment centers across the United Kingdom. Follow-up was conducted through linkages to Hospital Episode Statistics (HES), national death registries, and cancer registries; and currently available data from HES cover NHS hospital admissions in England and Scotland from April 1994 to February 2021.

In this study, our analyses were restricted to unrelated white British participants where the ancestry was determined through a combination of self-reports and principal component analysis of genotypes [17]. We excluded individuals with 1) missing genetic data; 2) inconsistent self-reported and genetic sex; 3) poor heterozygosity or high missingness; 4) aneuploid sex chromosomes; or 5) withdrawal of informed consent. CAD patients were identified based on a composite of self-reports in an interview with a trained nurse, and hospital admission records including both inpatient International Classification of Diseases (ICD-10, ICD-9) diagnosis codes, and the Office of Population Censuses and Surveys (OPCS-4) procedure codes [18].

We used phase three genotype data released by UKBB where the participants underwent genotyping with one of two closely related Affymetrix microarrays (UK BiLEVE Axiom Array or UK Biobank Axiom Array) for ~820,000 variants. Additional genotypes were imputed centrally using the 1000 Genomes and Haplotype Reference Consortium (HRC) reference panels, yielding ~93 million variants for each individual. We restricted the analysis to 2,994,054 autosomal variants with imputation quality score > 0.3, Hardy-Weinberg p-value > 1e-5, minor allele frequency (MAF) > 0.05, and genotyping missing rate < 0.01.

### Construction of pleiotropy-decomposed PRSs

#### Overview.

**Fig 1:**
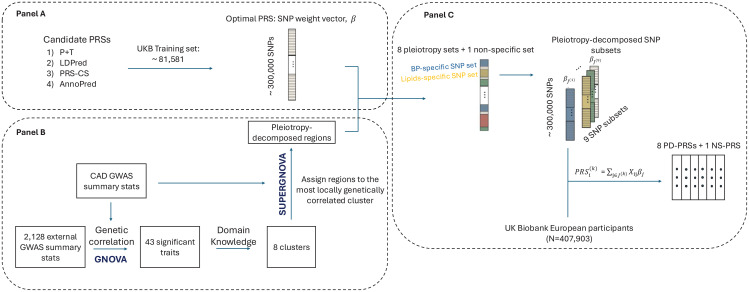
Construction of pleiotropy-decomposed polygenic risk scores (PD-PRS). By comparing four PRS methods using UKBB training data, we selected the best PRS with around 3 million SNPs for downstream decomposition (Panel **A)**. We selected diseases/traits that were genetically correlated with CAD from 2,128 GWAS summary statistics and grouped them into 8 pleiotropy clusters based on domain knowledge (Panel **B)**. SNPs in 2,353 independent LD blocks were then assigned to mutually exclusive SNP subsets including eight pleiotropy-decomposed subsets and one non-specific subset (others subset) be leveraging local genetic correlation analyses (Panel B and **C)**. The PD-PRSs were then calculated as the weighted sum of the SNPs within the pleiotropy-decomposed SNP subsets, weighted by the original effect sizes from the best CAD PRS (Panel **C)**.

We summarize the construction of PD-PRS in Fig 1. First, we identified 43 traits that were genetically correlated with CAD and classified them into 8 pleiotropy clusters (Fig 1B). Subsequently, we trained 130 candidate PRSs and selected the one with the best prediction performance for CAD (Fig 1A). Using region-level local genetic covariances, we further assigned SNPs included in the optimal PRS into 43 trait-specific groups and labeled these SNPs according to the corresponding pleiotropy clusters of traits (Fig 1B and 1C). SNPs that did not fit into any specific cluster were grouped into a ninth cluster. Finally, we calculated 9 PD-PRSs, each based on SNPs from one of the 9 clusters (Fig 1C).

#### Definition for pleiotropy clusters.

We defined pleiotropy clusters by leveraging the genetic correlations between CAD and traits using a two-step procedure. In the first step, we used GNOVA [19], a method estimating genetic correlations between phenotypes with consideration of biological annotations, to identify the diseases and traits that were genetically correlated with CAD by leveraging the large-scale CAD GWAS summary statistics (60,801 CAD cases and 123,504 controls) [4] and GWAS summary statistics for 2,128 diseases/traits. False discovery rate (FDR) correction was applied to select diseases/traits genetically correlated with CAD for cluster constructions [20]. In the second step, the 43 selected CAD-related diseases/traits were grouped into eight pleiotropy clusters from domain knowledge. To show the robustness of these domain knowledge-based pleiotropy clusters, we also built a genetic correlation matrix for these 43 selected CAD-related diseases/traits (Fig A in S1 Text). Each cell corresponded to the pairwise genetic correlation calculated between the corresponding traits using GNOVA [19], and the genetic correlations within each cluster and between clusters were compared.

#### Derivation of CAD PRS.

We considered four PRS methods, including P + T [21], LDPred [13], PRS-CS [15], and AnnoPred [14], to infer the effect sizes for SNPs. The one that had the best prediction accuracy for CAD was selected. P + T denotes the linkage disequilibrium (LD) clumping and p-value thresholding method, which was performed using PLINK version 1.90b (using the --clump flag) [21]. We varied the P value from 1.0, 0.5, 0.05, 5E-4, 5E-6, to 5E-8, and the $r^{2}$ value from 0.2, 0.4, 0.6, 0.8 to 1.0, resulting in a total of 30 combinations. LDPred [13], the auto version of PRS-CS [15], and AnnoPred [14] are all Bayesian approaches to infer the posterior mean effect size of each SNP from GWAS summary statistics while taking LD into account. For both LDPred and AnnoPred, we considered the tuning parameter, proportion of causal SNPs, within the set of {1.0, 0.3, 0.1, 0.03, 0.01, 3E-4, 1E-4, 3E-5, 1E-5, 3E-6, 1E-6} as suggested by the authors [13, 14]. In particular, since AnnoPred is a Bayesian framework that allows the incorporation of diverse types of genomic and epigenomic functional annotations to improve genetic risk prediction, we incorporated functional annotations [22–25], and trained the AnnoPred PRS by comparing results based on four different tiers of annotations as suggested previously [14].

Taken together, based on CAD GWAS summary statistics and the external LD reference panel of 503 European individuals from the 1000 Genomes Project phase III [26], we built 130 candidate CAD PRSs using these four methods with different tuning parameters. The best performing CAD PRS was selected based on the C-index in a Cox regression model via five-fold cross-validation with the disease status as outcome, the onset age as follow-up time, and the candidate PRS as the predictor in a randomly selected training subset of 81,581 European individuals from UKBB.

#### Partition of CAD PRS into pleiotropy-decomposed PRS.

We employed a region-based strategy to partition the best-performing CAD PRS into PD-PRSs. More specifically, we first divided the genome into 2,353 LD blocks (about 1.6 centimorgans on average) that were approximately independent of each other based on the 1000 Genomes European reference panel [26]. SUPERGNOVA [27, 28], a method to estimate genetic correlations for nearly LD-independent genome regions in a stable way, was subsequently used to calculate the local genetic covariances in each block between CAD and the 43 CAD-related diseases/traits. For each block, we assigned the SNPs in this region to the disease/trait with a significant genetic correlation (P < 0.05) and the strongest genetic covariance. Of note, the absolute value of genetic covariance was the measurement for local genetic relevance with little impact from local heritability. SNPs assigned to specific diseases/traits were labeled with the corresponding pleiotropy cluster we defined (See Definition for pleiotropy clusters). It is worth noting that, for some blocks, none of the 43 CAD-related diseases/traits demonstrated a significant local genetic correlation (P > 0.05) with CAD. Hence, we grouped the SNPs in these blocks together as one subset and tagged it as non-specific cluster. Through this process, we assigned around 3,000,000 variants into nine pleiotropy clusters, including eight pleiotropy-decomposed (PD) subsets and one non-specific subset which was named the ‘others PD cluster’. Of note, like many genetic data, e.g., tag SNPs, the assigning result is a proxy rather than an accurate representation of biology meaning. Finally, for each pleiotropy cluster, its corresponding pleiotropy-decomposed PRS (PD-PRS) was computed as the weighted sum of affected alleles of the SNPs within each pleiotropy-decomposed subset, weighted by the pre-trained weights from the top CAD PRS (See Derivation of CAD PRS).

### Statistical analyses

#### Phenome-wide association analysis (Phe-WAS) of pleiotropy-decomposed PRSs.

To evaluate whether the PD-PRSs could represent the corresponding pleiotropy cluster well, we first investigated the associations between these PD-PRSs and CAD-related traits/biomarkers. More specifically, 59 potential CAD-related traits/biomarkers from 14 biological categories were collected, and associations were evaluated between the PD-PRSs and binary and non-binary traits/biomarkers via logistic and linear regressions, respectively.

#### Identify subgroups among people with high CAD genetic risk.

We used our PD-PRSs to identify pleiotropy subgroups among individuals with high CAD genetic risk. Among those individuals with the top 5% overall CAD PRS, we selected members of pleiotropy subgroups as those who also ranked in the top 5% corresponding PD-PRSs across the entire study population. As a result, we assigned the individuals with the top 5% overall CAD PRS into nine subgroups that corresponded to nine PD-PRSs. These subgroups allowed for overlapping with some individuals in more than one subgroup and some subjects were not in any of the subgroups. High-risk individuals not present in specific subgroups were grouped into the subgroup-specific remaining high-risk set, and in the following analyses, we compared subgroup individuals with subjects in this remaining set.

We then calculated pleiotropy-based relative differences to investigate the specificity of these pleiotropy subgroups. Formally, the relative change of a quantitative trait for a specific group was calculated as (L_S_ - L_R_)/SD, where L_S_ was the average quantitative trait value for those individuals in a subgroup, L_R_ was that for the remaining individuals with high CAD PRS (top 5% CAD PRS), and SD was the standard deviation among individuals with high CAD PRS. Similarly, the relative change of a disease was calculated as (P_S_ - P_R_)/SD, where P_S_ and P_R_ were the prevalence of the disease in the pleiotropy subgroup and remaining individuals with high CAD PRS, respectively.

#### Interaction analyses.

We hypothesized that interactions between CAD-related risk factors and the overall CAD PRS can be attributed to certain PD-PRSs, and individuals in the subgroup defined by these PD-PRSs might be affected more by the corresponding interacting risk factor. Eight lifestyle-related traits, nineteen physical measurements, and two socioeconomic status (SES) factors were selected for this analysis (Table H in S1 Text). For lipids-related traits, we corrected the effects of medications and removed outliers with values beyond five standard deviations from the mean.

We adjusted the traits of interest for potential multicollinearity that could lead to false positive results in interaction tests. For continuous variables, we used linear regression models, and for binary variables, we employed logistic regression models to adjust for the effects of standardized age at recruitment, squared standardized age, sex, the top four principal components (PCs), body mass index (BMI), age-sex interactions, squared age-sex interactions, PD-PRSs, and trait-specific covariates (i.e., smoking for non-smoking traits, cholesterol-lowering medicine for lipid measures, and anti-hypertension medication for blood pressure measurements) (eq (1)). The residuals from equation (1) were then transformed using the rank-based inverse normal transformation (INT), and interactions between these transformed traits and PD-PRSs were evaluated (eq (2)). Specifically, we constructed two models—one including and one excluding the interaction term—and applied a likelihood ratio test. The null hypothesis posited that including the interaction term did not improve the model fit. Interactions were significant if the p-value was less than 0.05 after B-H FDR correction. Note that the residuals from the logistic regression differ from the original binary variables; therefore, the results for binary variables should be interpreted with caution.


$$\begin{array}{c} \begin{array}{c} Trait\sim PRS+ageatrecruitment+{ageatrecruitment}^{2}+sex+ageatrecruitment*sex+{ageatrecruitment}^{2}*sex\\ +PC1+PC2+PC3+PC4+BMI+trait.specific.covariates. \end{array} \end{array}$$ (1)

$$\begin{array}{c} CAD\sim PRS*INT.transformed.residualsfromeq(2)+ageatrecruitment+{ageatrecruitment}^{2}+sex+ageatrecruitment*sex\\ +{ageatrecruitment}^{2}*sex+PC1+PC2+PC3+PC4+BMI+trait.specific.covariates. \end{array}$$ (2)

Sensitivity analyses were performed to evaluate the robustness of our interaction analysis with respect to both covariate selection and trait transformation. In one analysis, we replaced the original covariate set with age at recruitment, sex, and the top four principal components, then re-examined the interactions. Separately, we conducted an independent analysis using residuals obtained from direct covariate adjustment without applying INT. These two approaches were carried out independently.

Moreover, we hypothesized that subgroups defined by PD-PRSs interacted with the same risk factor identified. We kept pairs that were significant in the interaction test, dichotomized continuous variables based on prior knowledge for absolute risk analysis, and assessed the impact of interactions among individuals with high genetic risk for CAD. Specifically, we explored the relative and absolute risk of interactions by comparing subgroups and remaining high-risk subjects. Hazard ratios (HRs) were used to evaluate the relative risk. We evaluated whether the interaction effect significantly differed from zero using the likelihood ratio test; a p-value below 0.05 indicated statistical significance. For absolute risk, we calculated the absolute risk reduction (ARR) for these traits in different PD-PRS subgroups, where the absolute risk (AR) was calculated as the proportion of CAD cases in a specific subgroup. Furthermore, to measure the potential benefits of improving lifestyles or lowering physical measurements in the subgroup compared to other high-risk individuals, we calculated the relative excess risk due to interaction (RERI) as $({ARR}_{subgroup}-{ARR}_{remain})/R_{00}$, where $R_{00}$ is the proportion of incident CAD cases among the remaining high-risk subjects not exposed to the harmful phenotype. We assessed the null hypothesis of no additive interaction using the Delta method [29] with a p-value below 0.05 as statistical significance.

All the interaction analyses were conducted using Cox proportional hazard regression models. Follow-up time began from the recruitment date and was censored at CAD onset, death, or February 5, 2021, whichever was earliest. For interaction analysis, we applied the B-H FDR-controlled correction for multiple comparisons with a significance threshold of 0.05 [20]. All statistical analyses were conducted using R (version 4.2).

## Simulation

To validate the plausibility of the PD-PRS framework, we conducted simulations for both GWAS and phenotypes. We simulated GWAS beta coefficients for four traits using HapMap 3 SNPs on chromosome 22 and genotype data from the 1000 Genomes European population. Specifically, we decomposed chromosome 22 into 47 nearly independent blocks based on the LD structure of the 1000 Genomes European genotype data, and then randomly selected 10 causal regions for each trait. Trait 1 served as the target trait, and three randomly chosen causal regions were designated as shared with the other three traits without overlap. We simulated local heritability under the assumption that the causal regions exhibit higher heritability. Furthermore, 10 regions—including three shared causal regions, two randomly selected unique causal regions for the trait of interest, and five non-causal regions—were designated as correlated regions between Trait 1 and each of the remaining three traits, and the genetic correlations for these regions were simulated. Local genetic correlations for non-correlated regions were assumed to be zero. Beta coefficients were simulated region by region across all four traits, incorporating both causality and local genetic correlations. Finally, random noise was added to the simulated beta coefficients, assuming a GWAS sample size of 50,000. Subsequently, we applied the PD-PRS framework to decompose the PRS for Trait 1 into four PD-PRSs: Trait 2-related, Trait 3-related, Trait 4-related, and others PD-PRSs.

For phenotype simulation, to generate Traits 2–4, we first calculated the genetic component of each trait as the weighted sum of genotype data, using the simulated beta coefficients as weights. The genotype data were obtained from unrelated White British subjects in the UKB. Next, we generated a non-genetic term drawn from a standard normal distribution. The final phenotype was calculated as the weighted sum of the genetic component, the non-genetic term and their interaction, with weights of 2, 1, and 0.3, respectively. For Trait 1, we incorporated the simulated values for Traits 2–4 along with their interactions with the corresponding PD-PRSs using weights of 1 and 0.3, respectively.

We then the genetic and phenotypic heterogeneity among UKB using the PD-PRSs and simulated phenotypes. Additionally, interaction analyses were performed.

## Results

### Population characteristics

After excluding 94,715 individuals according to the criteria, a total of 407,903 European participants (mean age: 56.91; male proportion: 45.94%) from UKBB were included in our study. Among these individuals, a total of 20,411 were diagnosed with CAD. The baseline characteristics of CAD and non-CAD subjects are summarized in Table 1. CAD cases showed a higher average age at recruitment, a larger proportion of males, elevated BMI values, a higher proportion of ever or current smokers, and a higher incidence of hypertension and diabetes. For our analyses, we randomly divided the subjects into a training set and a testing set with a ratio of 1:4, where the training set was mainly used for the selection of the best-performing CAD PRS.

**Table 1:** Demographic characteristics.

	CAD cases (N = 20,411)	Non-CAD controls (N = 387,492)
Age at recruitment^#^	61.8 (6.02)	56.66 (8.01)
Sex (Male)*	15,952 (78.15%)	171,443 (44.24%)
BMI (kg/m^2^)	29.04 (4.72)	27.33 (4.74)
Ever or current smoker	13,057 (64.38%)	171,528 (44.41%)
LDL (mmol/l)	3.03 (0.95)	3.6 (0.86)
HDL (mmol/l)	1.22 (0.32)	1.46 (0.38)
Cholesterol (mmol/l)	4.91 (1.26)	5.76 (1.12)
Triglycerides (mmol/l)	2.02 (1.15)	1.74 (1.02)
Hypertension	16,928 (82.94%)	208,650 (53.85%)
Diabetes	3,718 (18.22%)	15,168 (3.91%)

#: for continuous variables, mean (standard deviation); *: for binary variables, number (proportion); CAD: coronary artery disease; BMI: body mass index; LDL: low-density lipoprotein; HDL: high-density lipoprotein.

### Associations between PD-PRSs and CAD

Four PRS methods were considered in our study: P + T [21], LDPred [13], PRS-CS [15], and AnnoPred [14]. Specifically, AnnoPred is a genome-wide Bayesian method that leverages functional annotations in adjusting variant effects to better model genetic risk [14]. Our results suggest that AnnoPred provided the best predictive performance for CAD with C-index in the training set being 0.61 (95% CI, 0.60-0.62) (Table B in S1 Text). In total, 2,994,054 variants were included in the enhanced AnnoPred CAD PRS.

Before decomposing the CAD PRS into PD-PRSs, we first identified clusters by leveraging the pleiotropy of diseases/traits with CAD. As illustrated in Fig 1, by calculating the genetic correlations between 2,128 diseases/traits [4] and CAD, we selected 43 diseases/traits that were significantly genetically correlated with CAD. These 43 diseases/traits were then grouped into eight pleiotropy clusters, including the basic condition-related cluster, blood pressure (BP)-related cluster, non-CAD cardiovascular disease (CVD)-related cluster, immune system-related cluster, lipids-related cluster, obesity-related cluster, respiratory system-related cluster, and type 2 diabetes (T2D)-related cluster (Fig A and Table A in S1 Text). We first assigned indices to the selected GWAS summary statistics by grouping them according to trait or disease. For example, we labeled 2-hour glucose level (2hGlu) as Glucose 2117 and fasting glucose (FG) as Glucose 2118 to indicate that both GWASs pertained to glucose. Next, we selected biologically relevant indices for each cluster. Specifically, we grouped atrial fibrillation (AF), cerebrovascular disease, heart failure (HF), and valvular disease into cardiovascular disease (CVD) cluster [30]; combined blood pressure-related traits and hypertension into a blood pressure (BP) cluster; assigned lipids traits to a Lipid cluster; classified BMI within an obesity cluster; grouped lung cancer and smoking into a respiratory system cluster [31]; and combined glucose, HbA1c, and insulin with type 2 diabetes (T2D) into a T2D cluster. For the remaining traits/diseases, we grouped celiac disease, adhesive capsulitis of shoulder and primary biliary cirrhosis (PBC) into an immune-system-related cluster [32–34], while the remaining three formed a basic condition cluster. To evaluate the robustness of our cluster definitions, we conducted a sensitivity analysis using hierarchical clustering on the genetic correlation matrix with the number of clusters set to 5 (Fig B, panels A and B in S1 Text), 7 (Fig B, panels C and D in S1 Text), 9 (Fig B, panels E and F in S1 Text), and 15 (Fig B, panels G and H in S1 Text), respectively. When the clustering was restricted to nine groups, the hierarchical method broadly mirrored our domain knowledge-based definitions but exhibited reduced clarity—for example, parent longevity was co-clustered with obesity (Fig B, panel A in S1 Text). Limiting the analysis to five or seven clusters further compromised specificity: in the five-cluster case, Cluster 1 merged blood pressure and diabetes-related traits, making it difficult to assign a coherent label (Fig B, panel E in S1 Text). In contrast, expanding to fifteen clusters generated several nearly identical groups—Clusters 13–15 all captured diabetes signals—thereby undermining the practical value of finer subdivisions (Fig B, panel G in S1 Text). We then mapped high-risk individuals onto these hierarchical clusters and calculated relative phenotypic changes to evaluate heterogeneity (Fig B, panels B, D, F, and H in S1 Text). For every alternative clustering scheme, phenotypic differences were minimal and not readily distinguishable. Overall, purely data‑driven clustering fails to maintain both clear genetic partitioning and strong phenotypic separation. In contrast, our domain‐knowledge–driven clusters have better interpretability and preserve the largest relative changes across traits.

With these eight pleiotropy-defined clusters and one non-specific cluster, the CAD AnnoPred PRS was decomposed into nine complementary components, including one non-specific PRS (others PD-PRS). The associations between these decomposed PRSs and CAD were then evaluated in the UKBB dataset adjusting for age at recruitment, sex, and top 4 genetic PCs, with the results shown in Fig C in S1 Text. In particular, lipids-related PD-PRS showed the strongest association, with the hazard ratios (HRs) as 1.27 (95% CI: 1.26-1.29), which might be due to the largest number of SNPs involved except for the others PD-PRS (Fig C and Table C in S1 Text). In addition, we compared the HRs of PD-PRSs with the trait-PRSs generated directly from the GWAS summary statistics of the main trait in the corresponding cluster (Table D in S1 Text). From the comparison, we noticed that more than half of the eight PD-PRSs had higher HRs than the trait-PRSs, especially the lipids-related PD-PRS. The diminished magnitude of association of some PD-PRSs compared to the trait-PRSs was probably attributable to the predominant influence of lipids-related PD-PRS, as the increase of the power of one PD-PRS may lead to reduced associations of other PD-PRSs given that the sum of PD-PRSs was the overall CAD PRS. These results suggested that the PD-PRSs decomposed from CAD PRS were intrinsically not the same as the trait-PRSs developed directly from the trait-specific GWASs.

### Associations between PD-PRSs and CAD-related phenotypes

**Fig 2:**
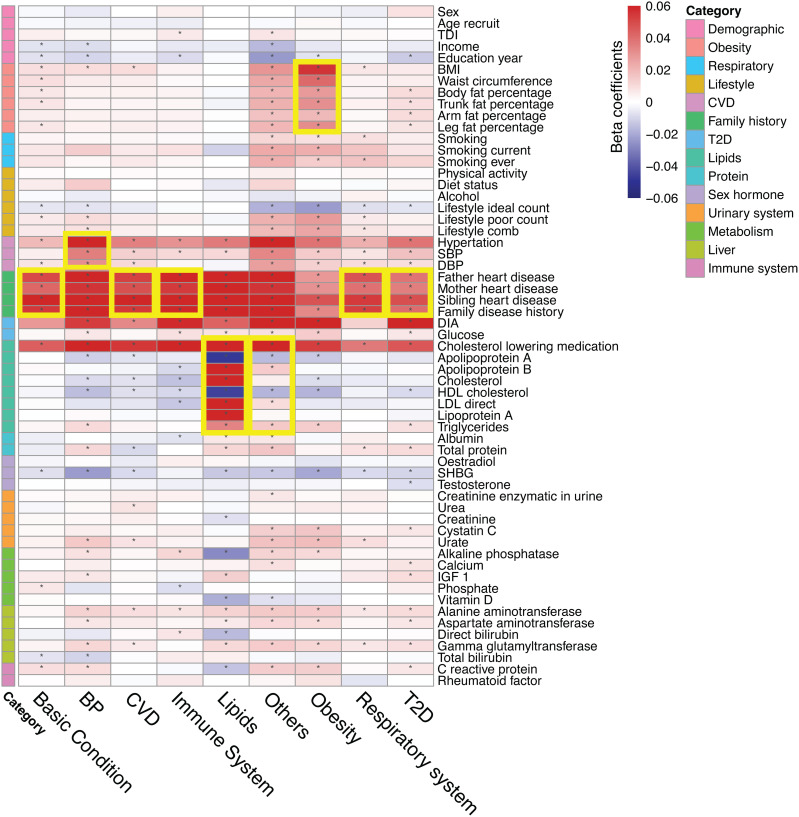
Correlations between phenotypes and PD-PRSs. We visualized the correlation coefficients between 59 phenotypes (rows) and nine PD-PRSs (columns), with the color representing the magnitude and direction of associations and the star indicating significant correlations (p-value < 0.05/(59*9)). The left legend bar is an annotation of the phenotype categories defined by domain knowledge. We used the yellow rectangular to highlight the phenotypes that showed the strongest association with each PD-PRS. Values included in this figure can be found in Table S5.

By analyzing the associations between our PD-PRSs and 59 CAD-relevant phenotypes from 14 categories, we found that our PD-PRSs could well represent the corresponding pleiotropy clusters (Fig 2 and Table E in S1 Text). For instance, the lipids-related PD-PRS was strongly correlated with lipids-related traits including cholesterol-lowering medication (P < 10^-20^), apolipoprotein A (P < 10^-20^), apolipoprotein B (P < 10^-20^), and others; the BP-related PD-PRS was mostly correlated with BP-related traits, including hypertension (P < 10^-20^), systolic blood pressure (SBP) (P < 10^-20^), and diastolic blood pressure (DBP) (P < 10^-20^); obesity-related PD-PRS was mostly correlated with BMI (P < 10^-20^), waist circumference (P < 10^-20^) and several fat percentage traits. Though not the strongest associations, respiratory system PD-PRS and T2D PD-PRS were significantly related to smoking behaviors and diabetes-related traits, respectively. Additionally, most PD-PRSs showed strong associations with CVD-related phenotypes, family history of heart diseases, and the use of cholesterol-lowering medications, while demonstrating weak or no associations with demographic variables, proteins, urinary system phenotypes, liver function phenotypes, and immune-related phenotypes. These findings suggest that PD-PRSs capture the primary risk signals of the overall CAD PRS and are closely linked to CAD-related functions.

### Relative differences of clinical factors in pleiotropy subgroups

**Fig 3:**
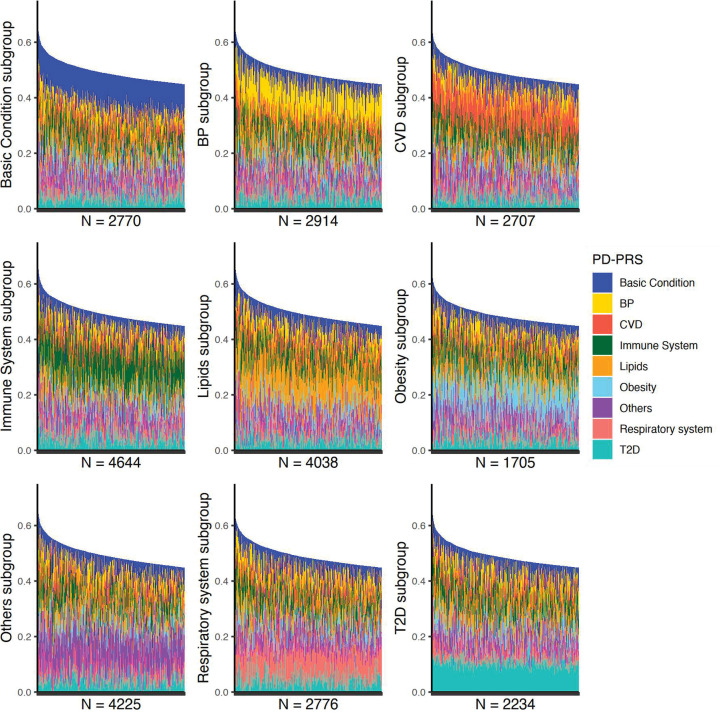
PD-PRS profiles in different pleiotropy subgroups. We assigned individuals with high CAD PRS into nine pleiotropy subgroups and visualized the contributions of PD-PRSs to the overall CAD PRS in each subgroup. Each subplot represents one subgroup with the sample size of each subgroup shown at the bottom. Each bar indicates one individual and the total height of the bar corresponds to this individual’s overall CAD PRS. Different colors represent the contributions of different PD-PRSs to the overall CAD PRS. Generally, individuals within a pleiotropy subgroup have a higher proportion of corresponding PD-PRS.

Based on PD-PRSs, we assigned the individuals with the top 5% CAD PRS into nine pleiotropy subgroups with each subject belonging to none, one, or multiple subgroups (See Methods). We explored the subgroup patterns, i.e., the number of subgroups the subject belonged to, and sample sizes of these patterns. A total of 250 distinct patterns were found, and 38 showed sample sizes of 100 or greater (Fig D in S1 Text). Of note, the majority of subjects were assigned to only one subgroup. Additionally, these subgroups had distinct distributions of PD-PRSs (Fig 3). In particular, individuals within a pleiotropy subgroup tended to have a higher genetic burden from traits involved in the corresponding cluster (Table F in S1 Text). For instance, among individuals in the lipids-related subgroup, the lipids-related PD-PRS contributed an average of 25.2% [standard deviation (sd) 0.07] of the global CAD PRS but only 10.1% [0.07] on average for the other individuals with high CAD genetic risk. Similar results were also found in other subgroups (24.1% [0.06] vs. 9.3% [0.07] for basic-condition-PD-PRS, 23.3% [0.05] vs. 9.3% [0.07] for BP-PD-PRS, 23.7% [0.05] vs. 9.3% [0.07] for CVD-PD-PRS, 23.8% [0.06] vs. 10.7% [0.07] for immune system-PD-PRS, 22.2% [0.05] vs. 8.7% [0.06] for obesity-PD-PRS, 24.6% [0.06] vs. 10.1% [0.07] for others PD-PRS, 23.6% [0.06] vs. 9.4% [0.07] for respiratory-system-PD-PRS, and 23% [0.05] vs. 9% [0.06] for T2D-PD-PRS). These results indicated a genetic heterogeneity among subjects with high CAD PRS, and such difference can be identified by the PD-PRSs.

**Fig 4:**
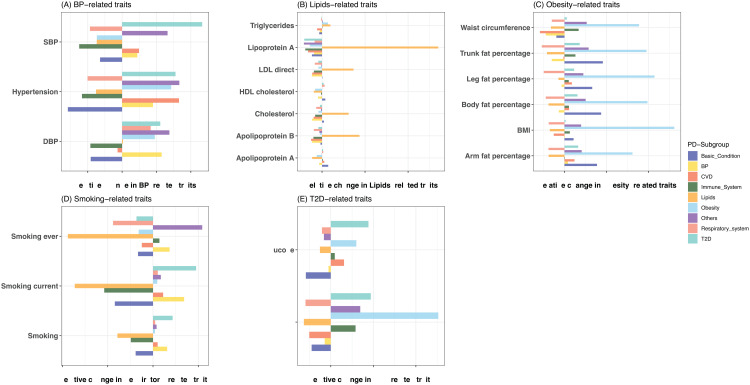
Relative differences of traits in pleiotropy subgroups. X-axis is the relative differences of **(A)** BP-related traits, **(B)** Lipids-related traits, **(C)** Obesity-related traits, **(D)** Smoking-related traits, and **(E)** T2D-related traits comparing individuals in pleiotropy subgroups versus the remaining high-risk individuals. The Y-axis presents the phenotypes considered. Different colors represent different pleiotropy subgroups. Values included in this figure can be found in Table S7.

More interestingly, individuals in different subgroups had distinct clinical features. For example, individuals in the lipids-related subgroup had significantly higher relative differences of lipids traits (e.g., lipoprotein(a), apolipoprotein B, LDL-cholesterol, etc.) compared to the remaining high-risk subjects; individuals in the obesity-related subgroup had significantly higher relative differences of obesity traits (BMI, waist circumference, body fat percentage, etc.) compared to the remaining subjects with high CAD PRS (Fig 4 and Table G in S1 Text). Similar patterns were observed for BP-related, Smoking-related, and T2D-related subgroups, although these did not show the highest relative changes. One possible explanation could be the strong pleiotropy across phenotypes. For example, previous studies have shown that T2D shares a substantial genetic component with obesity [35]. In line with these findings, the obesity-related subgroup demonstrated greater relative changes in T2D patients than the T2D-related subgroup. These together suggested genetic heterogeneity among individuals with high CAD genetic risk might lead to phenotypic differences. Through the application of PD-PRSs, it was feasible to distinguish subgroups with distinct clinical phenotypes due to the variations in their underlying genetic burdens.

A sensitivity analysis, in which the subgroup threshold was adjusted to 1% and 10%, yielded results similar to the main analysis (Fig E in S1 Text), thereby supporting the robustness of the subgroup definitions in this study.

### Interactions of PD-PRSs with clinical variables

We first selected interacting trait-PD-PRS pairs. Under the Cox regression model, we evaluated the interactions between 10 PRSs (nine PD-PRSs and the overall CAD PRS) with 29 traits of interest. The FDR-controlled p-values for interactions are presented in Table S9. Twenty-two significant trait-PRS pairs were found (underlined bold in Table I in S1 Text). Specifically, we identified 22 interaction pairs. Among these, three pairs involved blood pressure (BP)‐related PD-PRS—with sleep duration, forced vital capacity (FVC), and forced expiratory volume in one second (FEV1). Three pairs involved immune system–related PD-PRS—with frequent, infrequent, and current tobacco smoking. Additionally, three pairs involved lipids‐related PD-PRS—including current smoking, low‐density lipoprotein (LDL), and triglycerides. Two pairs involved obesity‐related PD-PRS (FVC and FEV1), while three pairs involved PD-PRSs related to others and the respiratory system—with frequent current tobacco smoking interacting with others PD-PRS, frequent current tobacco smoking with respiratory system–related PD-PRS, and current smoking with respiratory system–related PD-PRS. Finally, eight pairs involved interactions between eight distinct traits and the overall CAD PRS.

Two independent sensitivity analyses were performed. In one analysis, we adjusted for baseline covariates—age at recruitment, sex, and the top four principal components. Under this model, most interactions identified in the main analysis remained significant; however, the interaction frequent current smoking and the Others PD-PRS was no longer significant. Conversely, twenty additional interaction pairs (e.g., between systolic blood pressure and the BP‐related PD‐PRS) reached significance in this sensitivity analysis, suggesting that the main analysis may have produced slightly more conservative results (Table J in S1 Text).

In a separate analysis, we compared the use of INT with an analysis based directly on the residuals of 29 traits (using logit residuals for binary traits) in interactions with 10 PRSs. After B-H FDR control, 12 significant interaction pairs were identified (Table K in S1 Text), and all these pairs were also detected in our main analysis. This finding indicates that the INT approach provided greater statistical power compared to directly using the residuals, thereby justifying our chosen methodology.

We further assessed the interactions between selected traits and their corresponding subgroup of trait-interacting PD-PRS within subjects with high genetic risk for CAD through comparisons of relative risk and absolute risk (Fig F and Table L in S1 Text). The continuous variables were dichotomized into exposure and non-exposure based on the clinical normal ranges. Subgroup analyses did not reveal associations that were significantly stronger than those observed in the remaining high-risk subjects. Overall, our findings suggest that the study may be underpowered to detect statistical interaction effects among high‐risk subjects.

Furthermore, we assessed the effects of interactions on the absolute risk of CAD within the high-risk population. We highlighted results for two pairs including current smoking and respiratory system subgroup and high frequency of current smoking and respiratory system subgroup (Fig F, panels AA and panel AB, and Table L in S1 Text). In the Respiratory system subgroup, 12.9% of the excess CAD risk for current smokers was due to the interaction (Fig F, panel AB in S1 Text), and the proportion increased to 16% for heavy smokers (Fig F, panel AA in S1 Text). However, neither RERI was significantly different from 0, showing p-values of 5.42 × 10^-1^ and 5.06 × 10^-1^, respectively (Table L in S1 Text). Our findings further supported the heterogeneity among high-risk subjects. Additionally, these results suggested that smoking could potentially provide more benefits in mitigating the risk of CAD for individuals within corresponding genetic subgroups compared to others in the high-risk population. However, it is important to acknowledge that these interaction analyses among high-risk subjects were underpowered; studies with larger sample sizes are needed to confirm these findings.

## Simulation

Using simulated data, we first decomposed the PRS for Trait 1 into four PD-PRSs—Trait 2-related, Trait 3-related, Trait 4-related, and Others—across unelated White British UKB participants. Similarly, we classified high-risk subjects into PD-subgroups. Fig G, panel A in S1 Text displayed the stacked contributions of these PD-PRSs to the overall Trait PRS within each subgroup. Across four subgroups, the PD‑PRS corresponding to the subgroup dominated the PRS composition, suggesting that our decomposition can capture genetic heterogeneity. Next, we calculated the relative change in phenotypes 2–4 by comparing each subgroup against the remaining high‑risk subjects (Fig G, panel B in S1 Text). Subgroups exhibited pronounced, trait‑specific enrichments: Trait 2 subgroup showed 60.6% higher Trait 2 than the remaining high-risk subjects while the mean relative change for other subgroups was -13.6%; Trait 3 subgroup showed 77.2% higher Trait 3 while the mean for other subgroups was -21.7%; Trait 4 subgroup showed 67.6% higher Trait 4 while the other subgroups showed a mean of -19.4%. These large, positive shifts, in contrast to negative changes in other subgroups, suggests that the genetic heterogeneity uncovered by PD‑PRSs translates into clear phenotypic divergence. Finally, interaction models between the INT-transformed traits and their respective PD-PRSs returned coefficients near the true simulated effect of 0.3, with all interactions remaining statistically significant (Table M in S1 Text). Collectively, these results demonstrate our framework’s ability to detect both genetic substructure and corresponding phenotypic variation.

We further applied the SVD-based method of Chasman et al. (2020) [9] to the simulated data. Because their approach required independent SNPs as input, we first identified independent SNPs through LD pruning using data from the 1000 Genomes Project. By applying singular value decomposition (SVD) to the phenotype-beta matrix, we extracted three component PRSs (accounting for a cumulative variance of ≥ 80%) and one residual PRS. To elucidate the biological functions of each component, we examined the associations between the component PRSs and the simulated traits. The results indicated that Component 1 PRS was most strongly associated with Trait 4, Component 2 PRS with Trait 3, and Component 3 PRS with Trait 2 (Table N in S1 Text). Finally, we identified subgroups among high-risk subjects using our definition (top 5%) based on the residual and component PRSs. Genetic heterogeneity was evident across these subgroups where subgroup subjects showed higher contributions from the relevant component PRSs (Fig G, panel C in S1 Text). Phenotypic analysis of the component‐based subgroups (Fig G, panel D in S1 Text) revealed more modest trait enrichments than our PD‑PRS framework. Specifically, the Component 1 subgroup exhibited a 28.6% increase in Trait 4, the Component 2 subgroup a 33.0% increase in Trait 3, and the Component 3 subgroup a 27.5% increase in Trait 2. By contrast, PD‑PRS–defined subgroups showed substantially larger relative changes in their focal traits—67.6% for Trait 4, 60.6% for Trait 2, and 77.2% for Trait 3 (Fig G, panel B in S1 Text)—more than doubling the effect sizes observed with the SVD‐derived PRSs. Because our simulation was designed so that higher trait‐specific scores translate into higher phenotype values, these amplified shifts indicate more effective stratification. In summary, while the Chasman et al. [9] method reveals underlying genetic substructure, the PD‑PRS framework delivers markedly stronger genetic–phenotypic discrimination.

## Discussions

In this study, we constructed clinically meaningful pleiotropy clusters, and accordingly built robust CAD PD-PRSs to study the genetic and phenotypic heterogeneity among individuals with high genetic risk for CAD. In particular, through the application of Phe-WAS analyses, we showed the clinical interpretability of PD-PRSs. Additionally, pleiotropy subgroups built upon the PD-PRSs exhibited differentiation with each subgroup manifesting a distinct profile of relative differences in CAD-relevant clinical features. Besides, the BP-related-PD-PRS explained the interaction between CAD PRS and BP-related traits. This relationship was also found for individuals in the BP subgroup, where larger effects of blood pressure on CAD were found, further supporting the heterogeneity among high-risk subjects and also showing the prospective clinical utility of the genetic pleiotropy subgroups.

Our PD-PRSs were partially consistent with the recent work from Chasman et al. [9], where they decomposed the CAD PRS into T2D-related, lipids-related, and BP-related PRS components. However, our method advances in identifying genetic subgroups with more extreme phenotypes in simulations by introducing several significant advancements, offering a more refined decomposition of CAD PRS and providing a more comprehensive study into the utility of these decomposed PRS components. Firstly, in Chasmans’ work, they used a CAD PRS generated from the P + T method, where only 130 SNPs were included; while our study was based on an enhanced AnnoPred CAD PRS, which included millions of SNPs and was significantly more predictive. Secondly, in previous work, the SVD was used for the PRS decomposition, where a pre-constructed matrix of genetic effects of the SNPs across a panel of selected disease-related phenotypes was required. Due to the computational burden of SVD for big matrix, their framework could hardly be extended to the case with millions of SNPs, and their selections of CAD-related phenotypes were also subjective; both can lead to potentially more limited and unstable results. Our study adopted a region-based decomposition framework instead, which facilitated the sophisticated decomposition of a more advanced CAD PRS. In our study, the definitions of pleiotropy clusters were less subjective through scanning the genetic correlations between CAD and a large number of phenotypes. Furthermore, under our framework, PD-PRSs were built from mutually exclusive SNP subsets, and they sum up to the overall CAD PRS, which also improved the interpretability of the decomposed PRSs. In addition, while previous work considered the overall general population, our analyses targeted the population with the top 5% CAD PRS to explore the heterogeneity among people with high genetic risk of CAD.

Decomposition of the overall CAD PRS into PD-PRSs enabled us to uncover both genetic and phenotypic heterogeneity among individuals at high genetic risk for CAD. Our analysis revealed that most subjects with a high overall CAD PRS exhibited a dominant PD‐PRS (Figs C and D and Table G in S1 Text), suggesting that their genetic risk is largely driven by a single pathogenic pathway. This observation is consistent with previous studies showing that the large number of CAD-associated genes can be aggregated into a few core mechanisms—for instance, lipid metabolism, inflammation, and immune regulation [8,36,37]—and provide an aggregated framework for quantifying the contributions of distinct biological processes. Moreover, the unique subgroup patterns indicate that some CAD patients may be predominantly affected by a specific cluster of genetic factors with similar functions. This finding aligns with reports that have organized CAD risk genes into functional networks, such as the lipid metabolism network [38]. Furthermore, subjects classified into more than one subgroup—such as the 351 individuals who belonged simultaneously to both immune‐ and lipid‐related subgroups—exhibited patterns that mirror known links between these biological systems in modulating cardiovascular risk [39,40]. Collectively, these results support the utility of PD‐PRSs for dissecting the etiology of CAD.

Furthermore, the genetic heterogeneity we identified reflects a broad spectrum of phenotypic variation. In our analysis, PD‐PRSs were associated with a range of CAD‐related phenotypes, indicating that the genetic variants aggregated within the score capture distinct biological processes underlying CAD. This association with multiple phenotypes reinforces the biological relevance of PD‐PRSs and suggests that they may serve as effective molecular indicators for dissecting the mechanisms by which genetic factors contribute to CAD. Besides, when high‐risk subjects were stratified based on their PD‐PRS, certain subgroups exhibited more extreme phenotype values compared with other high‐risk individuals. In other words, among individuals already at high aggregated genetic risk, further stratification by PD‐PRSs revealed subgroups with distinct clinical profiles—suggesting that genetic enrichment in certain biological pathways may lead to different manifestations and potentially personalized treatment. This refined stratification aligns with emerging evidence that disaggregating polygenic risk into its functional components can improve risk prediction and help identify individuals who might benefit from certain therapies [41, 42]. These results suggested phenotypic heterogeneity among individuals with high CAD PRS, and such difference was caused by a genetic variety in the composition of PRS. Clinically, recognizing such genetic heterogeneity is critical. It opens the possibility of moving beyond “one‐size‐fits‐all” strategies by identifying distinct high‐risk subgroups that may respond differently to targeted interventions. In practical terms, incorporating PD‐PRS into risk models could enable earlier and more precise preventive measures—such as initiating statin therapy or lifestyle modifications in individuals whose risk is driven primarily by lipid metabolism abnormalities—thereby facilitating a more personalized approach to CAD management. Future studies will be essential to refine these genetic risk models and ensure that the benefits of personalized prevention strategies are equitably realized across diverse populations.

In addition, we observed several significant interactions between PD-PRSs and established risk factors for CAD. Notably, we identified significant interactions between respiratory system-related PD-PRSs and smoking-related traits, specifically current smoking status and high frequencies of smoking. Among individuals with an overall high CAD PRS, those with an elevated respiratory PD‐PRS appear to be especially susceptible to the cardiovascular harms of smoking. This subgroup’s genetic predisposition may amplify the deleterious effects of tobacco exposure, suggesting that smoking cessation could result in a more substantial reduction in CAD risk for these subjects compared with high‐risk individuals without such enrichment. The notion that smoking exacerbates the genetic risk for CAD through specific biological pathways further underscores the importance of targeted interventions for this high-risk subgroup. The interaction between smoking and genetic factors for CAD has been well-established, where it is demonstrated that harmful effects of smoking are more pronounced in individuals with genetic susceptibility to cardiovascular diseases [43, 44]. Gene- and pathway-level analyses further indicate that these interactions may be mediated by intensified inflammatory pathways [43,45]. Our results, therefore, not only corroborate these observations but also help pinpoint a distinct high‐risk genetic subgroup that may derive the greatest benefit from targeted smoking cessation interventions.

We also note several limitations of our study. First, even though we built PD-PRSs using non-overlapping SNPs, there were still correlations between some pairs of PD-PRSs (Fig H in S1 Text); which might be due to the shared downstream biological pathways of these exclusive subsets of genetic markers. However, it is worth mentioning that significant correlations were only observed between others PD-PRS and basic condition PD-PRS, suggesting the uncovered pleiotropic effects for non-specific SNPs. Furthermore, the negligible magnitude of inter correlations (-0.01 to 0.01) suggests their independence. Secondly, we used a high threshold of CAD risk when defining the subgroup (top 5%) to ensure the differences between subgroups and the remains were large enough to be detected. However, this cutoff inevitably decreased the sample size of subgroups, which might affect the statistical power of our study. A more balanced cutoff that optimally weighs statistical power against the subgroup distinctions should be explored in future studies. Thirdly, we defined the pleiotropy clusters based on a large-scale genetic correlation scan, which while effective in identifying major clusters, some important but small clusters might have been overlooked. Our others PD-PRS demonstrated a relatively large effect without being assigned to any specifically defined clusters, indicating the presence of such overlooked clusters. Our results are also affected by the GWAS sample sizes and genetic architecture of the traits. Fourthly, we used one-fifth of the study samples as the training set to select the optimal PRS and analyzed the PRS in the whole population, which may lead to a potential overfitting issue. However, given the limited sample sizes of the training set compared to that of the study, our conclusions should be held for subjects not included in the training dataset. Finally, this work served primarily as a discovery analysis for decomposing the overall CAD PRS into pleiotropy-informative components. To confirm the robustness and generalizability of these findings, additional replication in independent cohorts—including those of diverse ancestries—will be essential.

## Supporting information

S1 Text Table A. Selected 43 GWAS summary statistics and corresponding clusters. Table B. C-index of PRSs across four methods. Table C. Number of SNPs in pleiotropy-decomposed SNP subsets. Table D. Prediction performance of trait PRSs and PD-PRS for CAD. Table E. Correlations between phenotypes and PD-PRSs. Table F. Mean contributions of PD-PRSs to CAD PRS across subgroups. Table G. Relative changes of traits in pleiotropy subgroups. Table H. Traits for interaction analysis. Table I. False Discovery Rate-controlled p-values for interactions between 29 clinical traits and 10 PRSs. Table J. False Discovery Rate-controlled p-values for interactions between 29 clinical traits and PRS adjusted for baseline covariates. Table K. False Discovery Rate-controlled p-values for interactions between residuals of 29 clinical traits and PRS. Table L. Interactions among high-risk subjects. Table M. Simulations: interaction tests. Table N. Simulations: associations between component PRSs defined by Chasman et al. and simulated traits. **Fig A. Genetic correlations between traits.** Based on the genetic correlations with CAD, we selected 43 traits and grouped them into 8 clusters based on domestic knowledge. The genetic correlations between 43 selected traits were calculated by GNOVA. The correlation coefficients were shown above and the star indicated a significant genetic correlation after Bonferroni correction (p < 0.05/(43*43)). The left bar reflected the pathways we defined. **Fig B. Hierarchical clustering on genetic correlation matrix.** Sensitivity analysis of hierarchical clustering was conducted on the genetic correlation matrix derived from 43 selected traits using cluster numbers of 5 (panels A and B), 7 (panels C and D), 9 (panels E and F), and 15 (panels G and H). Panels A, C, E, and G display annotated heatmaps of the genetic correlations, whereas panels B, D, F, and H present the relative changes of the corresponding subgroups. When using 9 clusters (panel C), the resulting clusters were similar to those defined by our domain knowledge, although with reduced interpretability (e.g., parent longevity was grouped with obesity). With fewer clusters (5 and 7), specificity decreased, making it difficult to label the groups and interpret the results; for instance, in the 5-cluster scenario, Cluster 1 was related to both blood pressure and diabetes-related factors. In contrast, increasing the number of clusters to 15 resulted in many clusters with considerable similarity, such as Cluster 13–15 that were all related to diabetes. Additionally, we divided high-risk subjects into subgroups based on these hierarchical clusters and calculated relative changes to assess phenotypic heterogeneity (Fig S2 B, D, F, H). The phenotypic differences were hard to detect, especially for smoking-related traits. To align with our goal of generating interpretable PD-PRSs, we decided to define clusters using domain knowledge. **Fig C. Hazards ratios for CAD of overall CAD PRS, 8 PD-PRSs, and 1 NS-PRS.** We fitted Cox proportional hazards model for CAD and 10 PRSs (overall CAD PRS and 9 PD-PRSs). The resulted hazards ratios (HRs) were highly correlated with the number of SNPs included in the PRS in a positive way. For example, as the overall CAD PRS included the largest number of SNPs, it showed a much higher HR than other PRSs. Among the PD-PRSs, the Lipids-related PD-PRS had the largest number of SNPs and showed the highest HR. **Fig D. Number of subjects in 38 subgroup patterns with more than 100 individuals.** We categorized high-risk subjects into 250 subgroup patterns based on the subgroup assignments and only patterns with sample sizes of 100 or more were displayed in the Fig. The X-axis represented the subgroup pattern, and the Y-axis quantified the number of subjects in each pattern. Black bars denoted patterns where subjects were assigned to a single subgroup, and yellow bars were the ones with two subgroup assignments. Notably, patterns with single subgroup assignment showed larger number of subjects than other patterns. **Fig E. Sensitivity analysis for subgroup threshold.** We assessed the robustness of subgroup analysis by defining high-risk subjects with top 1% and 10% PRSs. The PD-PRS profiles were visualized in A for top 1% and B for 10%. The relative differences of traits in subgroups were plotted in C for top 1% and D for 10%. **Fig F. Interactions between PD-PRS subgroup and cholesterol, LDL, triglycerides, FVC, FEV1, PEF, current smoking and sleep duration.** We partitioned the individuals with high CAD PRS into four groups according to their PD-PRS (pathway-specific subgroups versus remaining) and dichotomized traits. The relative risk of three groups compared to the reference group was calculated as HR through Cox proportional hazards model (A-D, I-L, Q-T, Y, Z). The absolute risk in each group was calculated as the incident rate of CAD in the group, and the absolute risk reduction (ARR) reflected the reduction of absolute risk when lowering physical measurements or changing harmful behaviors. The relative excess risk due to interaction (RERI) indicated the proportion of excess risk in the PD-PRS subgroup that could be attributed to the interaction between PD-PRS and corresponding phenotypes (E-H, M-P, U-X, AA, AB). **Fig G. Simulation results.** To validate the plausibility of our framework, a simulation of GWASs and phenotype for UKB subjects was conducted. With the simulated data, we decomposed the PRS for trait 1 and conducted subgroup analyses using PD-PRS framework and method from Chasman et al. [9] With three PD and one non-specific SNPs subsets, we decomposed the PRS for trait 1 into 4 PD-PRSs: trait 2-related, trait 3-related, trait 4-related, and others (A and B). Using Chasman et al. method, the PRS was decomposed to three component PRSs and one residual PRS (C and D). (A and C) Contributions to the overall PRS. (B and D) The relative change of traits 2–4 comparing subgroups and the corresponding remaining subjects. Both our framework and Chasman et al. [9] can identify the genetic and phenotypic heterogeneity, and our method showed more extreme values in the relative change. **Fig H. Correlations between 9 PD-PRS.** We calculated the correlations between 9 PD-PRSs. The upper right triangle showed the extent of correlation coefficients and the yellow stars indicated significance (p < 0.05/(9*9)). The lower left triangle showed the p-values of the correlations. The others PD-PRS was significantly correlated with basic condition PD-PRS, suggesting uncovered functional SNPs within the others PD-PRS.
(DOCX)
