# Supplementary material for: Robust pleiotropy-decomposed polygenic scores identify distinct contributions to elevated coronary artery disease polygenic risk
Source: PLoS Comput Biol. 2025 Jun 26;21(6):e1013191. doi: 10.1371/journal.pcbi.1013191 (PMC12212871; doi:10.1371/journal.pcbi.1013191)
Supplement: S1 Text — Table A. Selected 43 GWAS summary statistics and corresponding clusters. Table B. C-index of PRSs across four methods. Table C. Number of SNPs in pleiotropy-decomposed SNP subsets. Table D. Prediction performance of trait PRSs and PD-PRS for CAD. Table E. Correlations between phenotypes and PD-PRSs. Table F. Mean contributions of PD-PRSs to CAD PRS across subgroups. Table G. Relative changes of traits in pleiotropy subgroups. Table H. Traits for interaction analysis. Table I. False Discovery Rate-controlled p-values for interactions between 29 clinical traits and 10 PRSs. Table J. False Discovery Rate-controlled p-values for interactions between 29 clinical traits and PRS adjusted for baseline covariates. Table K. False Discovery Rate-controlled p-values for interactions between residuals of 29 clinical traits and PRS. Table L. Interactions among high-risk subjects. Table M. Simulations: interaction tests. Table N. Simulations: associations between component PRSs defined by Chasman et al. and simulated traits. Fig A. Genetic correlations between traits. Based on the genetic correlations with CAD, we selected 43 traits and grouped them into 8 clusters based on domestic knowledge. The genetic correlations between 43 selected traits were calculated by GNOVA. The correlation coefficients were shown above and the star indicated a significant genetic correlation after Bonferroni correction (p < 0.05/(43*43)). The left bar reflected the pathways we defined. Fig B. Hierarchical clustering on genetic correlation matrix. Sensitivity analysis of hierarchical clustering was conducted on the genetic correlation matrix derived from 43 selected traits using cluster numbers of 5 (panels A and B), 7 (panels C and D), 9 (panels E and F), and 15 (panels G and H). Panels A, C, E, and G display annotated heatmaps of the genetic correlations, whereas panels B, D, F, and H present the relative changes of the corresponding subgroups. When using 9 clusters (panel C), the resulting clusters [file pcbi.1013191.s001.docx]

**Supplemental Tables**

**Table A. Selected 43 GWAS summary statistics and corresponding clusters.**

| Cluster | Index | Trait/Disease | Genetic correlation with CAD | Sample size |
| --- | --- | --- | --- | --- |
| Basic condition | Birth weight 201 | Birth weight | -0.10 | 24925 |
| Basic condition | Parent longevity 191 | Mothers age at death | 0.26 | 135638 |
| Basic condition | Parent longevity 193 | Fathers age at death | 0.24 | 120617 |
| BP | DBP 2104 | DBP | 0.78 | 69899 |
| BP | Hypertension 938 | Hypertensive diseases (excluding secondary) | 0.29 | 130347 |
| BP | Hypertension 1263 | Hypertension | 0.25 | 19273 |
| BP | MAP 2105 | MAP | 0.89 | 29182 |
| BP | PP 2106 | PP | 0.72 | 74079 |
| BP | SBP 2107 | SBP | 0.94 | 69909 |
| CVD | AF 1223 | Atrial fibrillation and flutter | 0.17 | 113847 |
| CVD | AF 1224 | Atrial fibrillation and flutter with reimbursement | 0.16 | 114659 |
| CVD | Cerebrovascular diseases 934 | Cerebrovascular diseases (Finngen) | 0.35 | 106736 |
| CVD | Cerebrovascular diseases 692 | STROKE | 0.35 | 55809 |
| CVD | Cerebrovascular diseases 257 | Ischaemic Stroke | 0.09 | 123476 |
| CVD | HF 1257 | All-cause Heart Failure | 0.19 | 67875 |
| CVD | HF 1259 | Heart failure, not strict | 0.19 | 63926 |
| CVD | Valvular diseases 1319 | Valvular operations | 0.22 | 21558 |
| CVD | Valvular diseases 1327 | Valvular heart disease including rheumatic fever | 0.20 | 298420 |
| Immune system | Celiac disease 196 | Celiac disease | 0.37 | 19273 |
| Immune system | Inflammation 1643 | Adhesive capsulitis of shoulder | 0.21 | 89278 |
| Immune system | PBC 217 | Primary Biliary Cirrhosis | 0.46 | 132236 |
| Lipids | HDL 2091 | HDL | -0.48 | 210967 |
| Lipids | HDL 2108 | HDL | -0.56 | 91419 |
| Lipids | LDL 2109 | LDL | 0.50 | 87041 |
| Lipids | LDL 2092 | LDL | 0.11 | 215196 |
| Lipids | Lipoprotein 815 | Disorders of lipoprotein metabolism and other lipidaemias | 0.28 | 114607 |
| Lipids | TC 798 | Pure hypercholesterolaemia | 0.29 | 123083 |
| Lipids | TC 2110 | TC | 0.39 | 91666 |
| Lipids | TG 2094 | TG | 0.42 | 211491 |
| Lipids | TG 2111 | TG | 0.54 | 88084 |
| Obesity | Obesity 2088 | Body Mass Index | 0.40 | 795640 |
| Obesity | Obesity 2089 | BMI | 0.54 | 234069 |
| Respiratory system | Lung cancer 192 | Lung cancer | 0.34 | 66975 |
| Respiratory system | Lung cancer 241 | Lung Cancer | 0.22 | 135638 |
| Respiratory system | Smoking 2125 | smoking | 0.47 | 74053 |
| Respiratory system | Smoking 2128 | smoking | 0.33 | 209915 |
| T2D | Glucose 2117 | 2hGlu | 0.27 | 15234 |
| T2D | Glucose 2118 | FG | 0.13 | 58074 |
| T2D | HBA1C 2100 | Hemoglobin A1(C) | 0.41 | 46368 |
| T2D | Insulin 2116 | FI | 0.41 | 51750 |
| T2D | Insulin 2102 | Insulin Resistance (HOMA-IR) | 0.46 | 46186 |
| T2D | T2D 2122 | Mahajan.NatGenet2018b.T2D.European | 0.58 | 898130 |
| T2D | T2D 2095 | Diagram_1000G_Diabetes_2017 | 0.73 | 158186 |

**Table B. C-index of PRSs across four methods.**

| Method | Parameter(s) | C-index | 95% CI |
| --- | --- | --- | --- |
| P_T | p=0.05, r2=0.8 | 0.59 | 0.58-0.60 |
| LDPred | p=0.001 | 0.51 | 0.50-0.52 |
| PRScs | - | 0.60 | 0.59-0.62 |
| AnnoPred | Tier2, p=0.003, pT | 0.61 | 0.60-0.62 |

**Table C. Number of SNPs in pleiotropy-decomposed SNP subsets.**

| Cluster | Number of SNPs |
| --- | --- |
| Others | 1,975,408 |
| Lipids | 187,582 |
| BP | 177,751 |
| Immune system | 137,792 |
| CVD | 122,306 |
| T2D | 117,092 |
| Basic condition | 94,079 |
| Respiratory system | 93,775 |
| Obesity | 88,270 |

**Table D. Prediction performance of trait PRSs and PD-PRS for CAD.**

| Trait PRS | HR (95% CI) | PD-PRS | HR (95% CI) |
| --- | --- | --- | --- |
| Mother age at death | 0.88 (0.87, 0.9) | Basic condition | 1.16 (1.14, 1.18) |
| Hypertension | 1.21 (1.2, 1.23) | BP | 1.19 (1.17, 1.21) |
| Cerebrovascular disease | 1.12 (1.1, 1.13) | CVD | 1.18 (1.17, 1.20) |
| PBC | 1.02 (1.01, 1.04) | Immune system | 1.24 (1.22, 1.25) |
| HDL | 0.86 (0.85, 0.87) | Lipids | 1.28 (1.26, 1.3) |
| BMI | 1.23 (1.21, 1.24) | Obesity | 1.27 (1.26, 1.29) |
| Ever-smoking | 1.08 (1.07, 1.11) | Respiratory system | 1.08 (1.06, 1.09) |
| T2D | 1.23 (1.21, 1.25) | T2D | 1.13 (1.11, 1.14) |

PBC: Primary biliary cirrhosis.

**Table E. Correlations between phenotypes and PD-PRSs.**

| **Phenotype** | **Basic Condition** | **BP** | **CVD** | **Immune System** | **Lipids** | **Others** | **Obesity** | **Respiratory system** | **T2D** |
| --- | --- | --- | --- | --- | --- | --- | --- | --- | --- |
| Sex | 0 (5.1e-01)^*^ | -0.01 (9.4e-02) | 0 (8.8e-01) | 0 (1.3e-01) | 0 (9.0e-01) | 0 (1.0e+00) | 0 (2.8e-01) | 0 (1.8e-01) | 0.01 (1.0e-02) |
| Age Recruit | 0 (1.7e-01) | 0 (2.5e-02) | 0 (1.2e-02) | 0 (3.5e-03) | 0 (8.5e-01) | 0 (2.7e-01) | 0 (1.8e-01) | 0 (1.7e-01) | 0 (9.2e-01) |
| TDI | 0 (2.1e-03) | 0 (9.3e-02) | 0 (1.1e-01) | 0.01 (7.0e-05) | 0 (9.2e-01) | 0.01 (3.4e-06) | 0 (7.8e-02) | 0 (2.0e-01) | 0 (3.9e-01) |
| Income | -0.01 (4.1e-05) | -0.01 (1.7e-06) | 0 (1.3e-01) | -0.01 (6.5e-04) | 0 (6.0e-03) | -0.02 (5.6e-22) | -0.01 (8.0e-04) | 0 (1.6e-01) | -0.01 (2.2e-03) |
| Education Year | -0.01 (6.3e-06) | -0.01 (4.1e-10) | 0 (2.0e-03) | -0.01 (1.5e-07) | 0 (2.6e-01) | -0.02 (1.4e-51) | -0.01 (1.3e-05) | 0 (4.2e-02) | -0.01 (1.1e-14) |
| BMI | 0.01 (7.3e-09) | 0.01 (1.4e-09) | 0.01 (1.7e-09) | 0 (1.4e-02) | 0 (7.3e-01) | 0.03 (9.9e-79) | 0.06 (1.2e-277) | 0.01 (1.0e-05) | 0.01 (1.6e-04) |
| Body fat percentage | 0.01 (1.4e-05) | 0 (6.4e-03) | 0 (6.0e-03) | 0 (1.7e-02) | 0 (1.9e-01) | 0.02 (8.1e-40) | 0.03 (4.1e-71) | 0 (1.9e-02) | 0.01 (5.5e-10) |
| Trunk fat percentage | 0.01 (3.8e-06) | 0 (2.4e-02) | 0 (2.3e-02) | 0 (4.4e-02) | 0 (2.6e-02) | 0.02 (4.7e-43) | 0.03 (8.4e-84) | 0 (3.7e-03) | 0.01 (6.2e-09) |
| Arm fat percentage | 0.01 (4.8e-04) | 0 (6.0e-03) | 0 (9.6e-03) | 0 (1.3e-02) | 0 (6.0e-01) | 0.02 (4.7e-27) | 0.02 (3.4e-35) | 0 (1.7e-01) | 0.01 (7.3e-10) |
| Leg fat percentage | 0.01 (2.3e-05) | 0 (4.5e-03) | 0.01 (1.5e-03) | 0 (8.3e-03) | 0 (6.5e-01) | 0.02 (2.2e-37) | 0.03 (7.0e-88) | 0 (5.3e-02) | 0.01 (1.3e-06) |
| Waist circumference | 0.01 (1.9e-08) | 0.01 (2.4e-04) | 0.01 (8.9e-04) | 0 (1.5e-01) | 0 (8.1e-01) | 0.02 (4.1e-53) | 0.04 (4.1e-152) | 0 (2.1e-02) | 0.01 (4.1e-04) |
| Smoking | 0 (2.4e-02) | 0 (6.1e-02) | 0 (4.6e-02) | 0 (1.5e-01) | 0 (3.4e-01) | 0.01 (3.8e-13) | 0.01 (1.6e-07) | 0.01 (1.1e-07) | 0.01 (1.1e-03) |
| Smoking current | 0.01 (2.0e-01) | 0.01 (8.8e-03) | 0.01 (2.4e-01) | 0.01 (1.2e-01) | -0.01 (4.1e-02) | 0.02 (5.9e-06) | 0.02 (1.5e-05) | 0.02 (1.9e-03) | 0.01 (1.6e-01) |
| Smoking ever | 0.01 (2.3e-02) | 0 (3.5e-01) | 0.01 (4.8e-02) | 0 (3.1e-01) | 0 (9.6e-01) | 0.02 (2.0e-12) | 0.01 (9.0e-06) | 0.02 (1.4e-07) | 0.01 (3.8e-04) |
| Physical activity | 0 (3.0e-01) | 0 (6.1e-01) | 0 (2.9e-01) | 0 (3.7e-01) | 0 (4.1e-01) | 0.01 (7.9e-04) | 0 (5.0e-01) | 0 (6.4e-02) | 0 (3.7e-01) |
| Diet status | 0.01 (1.9e-01) | 0.01 (1.3e-03) | 0 (8.9e-01) | 0 (7.2e-01) | -0.01 (1.9e-01) | 0.01 (9.9e-03) | 0 (8.5e-01) | 0 (7.4e-01) | 0 (4.6e-01) |
| Alcohol | 0 (2.8e-01) | 0 (1.6e-03) | 0 (5.1e-01) | 0 (9.3e-01) | 0 (1.3e-01) | -0.01 (1.2e-04) | 0 (2.1e-02) | 0 (6.9e-03) | 0 (3.4e-01) |
| Lifestyle ideal count | -0.01 (8.1e-05) | -0.01 (1.8e-05) | -0.01 (1.0e-03) | 0 (3.1e-01) | 0 (6.7e-01) | -0.02 (2.1e-31) | -0.02 (1.5e-49) | -0.01 (8.4e-07) | -0.01 (5.0e-05) |
| Lifestyle poor count | 0.01 (5.4e-05) | 0.01 (3.8e-10) | 0.01 (2.0e-04) | 0 (2.3e-03) | 0 (1.1e-01) | 0.02 (1.0e-43) | 0.03 (9.9e-70) | 0.01 (6.6e-05) | 0 (6.9e-03) |
| Lifestyle comb | 0.01 (1.2e-04) | 0.01 (6.7e-08) | 0.01 (5.7e-04) | 0 (3.2e-01) | 0 (4.3e-01) | 0.02 (6.6e-34) | 0.03 (4.3e-58) | 0.01 (9.6e-07) | 0.01 (1.3e-04) |
| Hypertension | 0.02 (1.5e-09) | 0.08 (2.8e-153) | 0.03 (4.9e-28) | 0.03 (6.2e-22) | 0.04 (1.5e-30) | 0.08 (4.8e-154) | 0.04 (1.1e-34) | 0.02 (3.6e-12) | 0.04 (1.5e-33) |
| SBP | 0.01 (1.6e-03) | 0.04 (8.1e-103) | 0.01 (1.3e-16) | 0.01 (1.1e-10) | 0.01 (3.1e-11) | 0.03 (3.1e-89) | 0.01 (1.0e-18) | 0.01 (3.7e-06) | 0.02 (5.3e-26) |
| DBP | 0.01 (6.1e-06) | 0.03 (3.5e-79) | 0.01 (2.5e-10) | 0 (4.7e-02) | 0 (7.3e-02) | 0.03 (5.7e-60) | 0.01 (1.4e-15) | 0.01 (1.2e-09) | 0.01 (3.6e-08) |
| Father heart disease | 0.05 (6.9e-48) | 0.06 (7.7e-71) | 0.05 (1.1e-51) | 0.07 (9.3e-98) | 0.1 (1.2e-166) | 0.06 (1.1e-76) | 0.03 (1.4e-16) | 0.05 (1.1e-42) | 0.04 (1.7e-28) |
| Mother heart disease | 0.04 (2.5e-25) | 0.06 (4.2e-43) | 0.05 (1.2e-32) | 0.05 (4.5e-41) | 0.07 (2.7e-65) | 0.06 (1.4e-43) | 0.03 (5.5e-14) | 0.04 (3.4e-22) | 0.04 (1.3e-18) |
| Sibling heart disease | 0.06 (4.3e-28) | 0.08 (6.3e-42) | 0.06 (4.9e-28) | 0.08 (1.6e-46) | 0.1 (2.4e-69) | 0.09 (2.3e-50) | 0.05 (1.4e-16) | 0.05 (4.7e-21) | 0.05 (2.3e-17) |
| Family disease history | 0.05 (3.9e-67) | 0.07 (4.2e-105) | 0.06 (6.9e-68) | 0.08 (2.4e-125) | 0.1 (2.9e-204) | 0.07 (1.7e-115) | 0.03 (4.1e-25) | 0.05 (8.1e-62) | 0.04 (9.3e-38) |
| DIA | 0.03 (1.3e-04) | 0.05 (7.3e-13) | 0.03 (1.2e-05) | 0.06 (7.7e-15) | 0.04 (8.8e-09) | 0.09 (3.4e-31) | 0.1 (3.9e-40) | 0.01 (8.4e-02) | 0.06 (5.4e-16) |
| Glucose | 0 (1.0e-01) | 0.01 (2.1e-05) | 0 (1.1e-01) | 0.01 (6.8e-07) | 0.01 (1.6e-06) | 0.01 (6.7e-07) | 0.01 (4.3e-17) | 0 (2.1e-01) | 0.01 (2.2e-05) |
| Cholesterol lowering medication | 0.04 (1.6e-26) | 0.08 (1.1e-82) | 0.06 (9.0e-41) | 0.07 (1.8e-65) | 0.22 (0.0e+00) | 0.11 (1.2e-162) | 0.05 (2.1e-37) | 0.04 (1.1e-18) | 0.05 (2.2e-29) |
| Apolipoprotein A | 0 (6.8e-01) | -0.01 (7.2e-12) | -0.01 (3.4e-05) | -0.01 (1.7e-03) | -0.05 (8.4e-179) | -0.02 (1.8e-21) | -0.01 (8.3e-14) | 0 (1.1e-01) | -0.01 (4.0e-04) |
| Apolipoprotein B | 0 (1.7e-01) | 0 (4.5e-01) | 0 (5.1e-02) | -0.01 (1.2e-08) | 0.17 (0.0e+00) | 0.01 (1.3e-18) | 0 (3.4e-01) | 0 (2.1e-01) | 0 (4.9e-01) |
| Cholesterol | 0 (4.9e-01) | -0.01 (2.0e-07) | -0.01 (1.2e-05) | -0.01 (1.2e-19) | 0.12 (0.0e+00) | 0.01 (1.5e-03) | -0.01 (3.3e-07) | -0.01 (8.1e-04) | 0 (2.0e-02) |
| HDL cholesterol | 0 (8.0e-02) | -0.02 (9.4e-20) | -0.01 (4.9e-06) | -0.01 (1.5e-08) | -0.05 (1.1e-161) | -0.02 (7.4e-25) | -0.02 (8.8e-26) | 0 (2.9e-02) | -0.01 (8.6e-08) |
| LDL direct | 0 (5.6e-01) | -0.01 (1.1e-04) | -0.01 (2.3e-04) | -0.01 (3.5e-16) | 0.14 (0.0e+00) | 0.01 (8.9e-10) | -0.01 (7.2e-04) | -0.01 (1.3e-03) | 0 (9.1e-02) |
| Lipoprotein A | 0 (7.3e-01) | 0 (3.0e-01) | 0 (3.6e-01) | 0 (3.5e-01) | 0.25 (0.0e+00) | 0 (1.6e-01) | 0 (5.2e-01) | 0 (9.3e-01) | 0 (1.1e-01) |
| Triglycerides | 0 (4.4e-02) | 0.01 (3.1e-10) | 0 (3.1e-02) | 0 (5.9e-01) | 0.03 (3.9e-100) | 0.02 (7.4e-21) | 0.01 (4.6e-18) | 0 (5.5e-01) | 0.01 (2.1e-07) |
| Albumin | 0 (1.2e-02) | 0 (6.8e-01) | 0 (2.5e-02) | -0.01 (3.6e-05) | 0.01 (6.5e-05) | 0.01 (7.5e-05) | 0 (2.3e-01) | 0.01 (1.0e-03) | 0 (7.7e-01) |
| Total protein | 0 (8.1e-03) | 0.01 (1.7e-10) | -0.01 (4.4e-08) | 0 (9.0e-01) | 0.01 (3.8e-11) | 0.02 (8.5e-21) | 0 (3.6e-02) | 0.01 (1.6e-06) | 0.01 (2.8e-09) |
| Oestradiol | 0 (4.7e-01) | 0 (7.6e-01) | -0.01 (2.1e-01) | 0 (2.5e-01) | 0 (4.9e-01) | 0 (5.2e-01) | -0.01 (1.2e-01) | 0 (5.9e-01) | 0 (5.3e-01) |
| SHBG | -0.01 (5.3e-06) | -0.02 (8.0e-44) | -0.01 (2.7e-07) | 0 (5.9e-02) | -0.01 (1.3e-14) | -0.01 (6.3e-14) | -0.02 (9.7e-35) | -0.01 (9.0e-08) | -0.01 (2.0e-09) |
| Testosterone | 0 (7.9e-01) | 0 (6.1e-01) | 0 (9.1e-02) | 0 (2.0e-02) | 0 (1.3e-02) | 0 (7.5e-02) | 0 (1.1e-01) | 0 (9.3e-01) | -0.01 (2.0e-06) |
| Creatinine enzymatic in urine | 0 (3.0e-01) | 0 (4.0e-02) | 0.01 (5.5e-04) | 0 (5.0e-03) | 0 (7.4e-02) | 0.01 (3.1e-05) | 0 (1.7e-01) | 0.01 (4.8e-04) | 0 (3.4e-01) |
| Urea | 0 (8.4e-02) | 0 (2.0e-01) | 0.01 (2.0e-06) | 0 (9.6e-01) | 0 (2.0e-01) | 0 (7.3e-03) | 0.01 (3.5e-04) | 0 (4.4e-03) | 0 (7.0e-01) |
| Creatinine | 0 (9.1e-02) | 0 (7.6e-02) | 0 (1.9e-03) | 0 (4.3e-01) | -0.01 (2.7e-06) | 0 (7.1e-01) | 0 (7.2e-01) | 0 (5.3e-01) | 0 (8.7e-01) |
| Cystatin C | 0 (2.1e-01) | 0.01 (6.7e-04) | 0 (1.9e-01) | 0.01 (5.7e-04) | 0 (3.6e-01) | 0.01 (1.3e-16) | 0.02 (9.8e-23) | 0 (4.8e-03) | 0.01 (7.3e-06) |
| Urate | 0 (6.9e-03) | 0.02 (8.4e-21) | 0.01 (6.6e-07) | 0 (2.2e-02) | 0 (3.5e-02) | 0.02 (6.4e-26) | 0.02 (4.3e-30) | 0.01 (1.2e-08) | 0 (1.7e-02) |
| Alkaline phosphatase | 0 (1.0e-02) | 0.01 (1.3e-07) | 0 (4.5e-02) | 0.01 (1.6e-12) | -0.03 (2.6e-65) | 0.01 (1.3e-14) | 0.01 (1.7e-10) | 0 (6.8e-02) | 0 (1.9e-02) |
| Calcium | 0 (9.1e-01) | 0.01 (2.7e-03) | 0 (5.4e-01) | 0 (6.2e-02) | 0 (2.0e-02) | 0.01 (1.9e-07) | 0 (4.0e-01) | 0 (1.3e-02) | 0.01 (6.4e-06) |
| IGF 1 | 0.01 (2.6e-04) | 0.01 (9.6e-06) | 0 (8.4e-02) | 0 (7.6e-02) | 0.01 (1.7e-16) | 0 (3.6e-01) | 0 (1.5e-01) | 0.01 (9.9e-05) | 0.01 (4.0e-09) |
| Phosphate | 0.01 (1.8e-05) | -0.01 (1.8e-04) | 0 (6.3e-01) | -0.01 (2.0e-06) | 0 (4.4e-01) | 0 (7.6e-01) | 0 (7.2e-02) | 0 (8.5e-01) | 0 (4.3e-03) |
| Vitamin D | 0 (3.7e-01) | 0 (7.0e-01) | 0 (4.5e-01) | 0 (2.5e-01) | -0.02 (1.2e-30) | -0.01 (5.8e-06) | -0.01 (7.3e-04) | 0 (7.2e-01) | 0 (6.6e-01) |
| Alanine aminotransferase | 0 (6.6e-02) | 0.01 (1.5e-14) | 0.01 (1.9e-08) | 0.01 (1.7e-06) | 0.01 (8.4e-14) | 0.01 (6.0e-20) | 0.01 (4.2e-19) | 0.01 (2.4e-05) | 0.01 (8.4e-10) |
| Aspartate aminotransferase | 0 (1.0e+00) | 0.01 (3.0e-05) | 0.01 (4.6e-04) | 0 (3.4e-02) | 0.01 (7.4e-05) | 0.01 (1.0e-11) | 0.01 (1.0e-09) | 0 (7.0e-02) | 0.01 (9.6e-06) |
| Direct bilirubin | 0 (3.3e-02) | -0.01 (1.2e-03) | 0 (2.9e-01) | 0.01 (2.4e-05) | -0.02 (2.8e-20) | 0 (5.4e-01) | 0 (8.9e-01) | 0 (9.1e-01) | 0 (4.6e-01) |
| Gamma glutamyltransferase | 0 (2.1e-02) | 0.01 (7.0e-13) | 0.01 (8.3e-05) | 0 (5.3e-01) | 0.01 (2.0e-10) | 0.01 (3.4e-19) | 0.01 (9.1e-13) | 0.01 (3.3e-05) | 0.01 (1.0e-08) |
| Total bilirubin | -0.01 (3.8e-05) | -0.01 (8.0e-11) | 0 (4.6e-01) | 0.01 (4.5e-04) | 0 (2.5e-03) | 0 (3.1e-01) | 0 (1.1e-02) | 0 (9.4e-01) | 0 (4.5e-01) |
| C reactive protein | 0.01 (3.7e-08) | 0.01 (3.8e-09) | 0 (9.9e-01) | 0 (2.6e-01) | -0.01 (8.0e-18) | 0.01 (1.0e-17) | 0.01 (5.3e-17) | 0 (2.8e-01) | 0.01 (3.6e-05) |
| Rheumatoid Factor | 0 (8.3e-01) | 0.01 (3.4e-01) | 0 (8.2e-01) | 0.01 (2.3e-01) | 0 (9.1e-01) | 0 (5.7e-01) | 0.01 (1.6e-01) | -0.01 (2.4e-01) | 0 (9.4e-01) |

*: Beta coefficients (P-value); BP: blood pressure; CVD: cardiovascular disease; T2D: type 2 diabetes; TDI: Townsend deprivation index; BMI: body mass index; SBP: systolic blood pressure; DBP: diastolic blood pressure; DIA: diabetes.

**Table F. Mean contributions of PD-PRSs to CAD PRS across subgroups.**

| **Subgroup\PD-PRS** | **Basic Condition** | **BP** | **CVD** | **Immune system** | **Lipids** | **Obesity** | **Others** | **Respiratory system** | **T2D** |
| --- | --- | --- | --- | --- | --- | --- | --- | --- | --- |
| Basic Condition | 24.1% (0.06)^*^ | 9% (0.06) | 9.1% (0.07) | 10.7% (0.07) | 10.2% (0.07) | 8.4% (0.06) | 10.2% (0.07) | 9.2% (0.06) | 9.1% (0.06) |
| Basic Condition_Remain | 9.3% (0.07) | 11% (0.08) | 10.8% (0.08) | 13.1% (0.08) | 12.5% (0.09) | 9.7% (0.07) | 12.6% (0.09) | 10.9% (0.08) | 10.2% (0.07) |
| BP | 9.2% (0.07) | 23.3% (0.05) | 9.2% (0.07) | 10.6% (0.07) | 10.1% (0.07) | 8.8% (0.06) | 10.2% (0.07) | 9.4% (0.06) | 9.2% (0.06) |
| BP_Remain | 10.9% (0.08) | 9.3% (0.07) | 10.8% (0.08) | 13.1% (0.08) | 12.5% (0.09) | 9.6% (0.07) | 12.6% (0.09) | 10.9% (0.08) | 10.2% (0.07) |
| CVD | 9.1% (0.07) | 9.4% (0.07) | 23.7% (0.05) | 11% (0.07) | 10.2% (0.07) | 8.5% (0.06) | 10.1% (0.07) | 9.3% (0.07) | 8.9% (0.06) |
| CVD_Remain | 10.9% (0.08) | 10.9% (0.08) | 9.3% (0.07) | 13% (0.08) | 12.5% (0.09) | 9.6% (0.07) | 12.6% (0.09) | 10.9% (0.08) | 10.2% (0.07) |
| Immune System | 9.4% (0.07) | 9.4% (0.07) | 9.4% (0.07) | 23.8% (0.06) | 10% (0.07) | 9.2% (0.06) | 10.1% (0.07) | 9.6% (0.07) | 9.2% (0.07) |
| Immune System_Remain | 11% (0.08) | 11.1% (0.08) | 10.9% (0.08) | 10.7% (0.07) | 12.7% (0.09) | 9.6% (0.07) | 12.8% (0.09) | 11% (0.08) | 10.3% (0.07) |
| Lipids | 9.2% (0.07) | 9.3% (0.07) | 9.3% (0.07) | 10.3% (0.07) | 25.2% (0.07) | 8.6% (0.06) | 10% (0.07) | 9.2% (0.06) | 8.9% (0.06) |
| Lipids_Remain | 11% (0.08) | 11% (0.08) | 10.9% (0.08) | 13.3% (0.09) | 10.1% (0.07) | 9.7% (0.07) | 12.7% (0.09) | 11% (0.08) | 10.3% (0.08) |
| Obesity | 9% (0.07) | 9.5% (0.07) | 9.2% (0.07) | 11% (0.07) | 10.4% (0.08) | 22.2% (0.05) | 10.4% (0.07) | 9.4% (0.07) | 8.9% (0.06) |
| Obesity_Remain | 10.8% (0.08) | 10.9% (0.08) | 10.8% (0.08) | 13% (0.08) | 12.4% (0.09) | 8.7% (0.06) | 12.4% (0.09) | 10.9% (0.08) | 10.2% (0.07) |
| Others | 9.4% (0.07) | 9.3% (0.07) | 9.2% (0.07) | 10.5% (0.07) | 9.9% (0.07) | 8.8% (0.06) | 24.6% (0.06) | 9.4% (0.07) | 9% (0.06) |
| Others_Remain | 11% (0.08) | 11.1% (0.08) | 10.9% (0.08) | 13.3% (0.09) | 12.7% (0.09) | 9.7% (0.07) | 10.1% (0.07) | 11% (0.08) | 10.3% (0.08) |
| Respiratory system | 9.3% (0.07) | 9.3% (0.07) | 9.5% (0.07) | 10.7% (0.07) | 10% (0.07) | 8.5% (0.06) | 10.3% (0.07) | 23.6% (0.06) | 8.8% (0.06) |
| Respiratory system_Remain | 10.9% (0.08) | 11% (0.08) | 10.8% (0.08) | 13.1% (0.08) | 12.5% (0.09) | 9.6% (0.07) | 12.5% (0.09) | 9.4% (0.07) | 10.3% (0.07) |
| T2D | 9.2% (0.07) | 9.6% (0.07) | 9.1% (0.07) | 11.1% (0.07) | 10% (0.07) | 8.7% (0.06) | 10.2% (0.07) | 9.2% (0.06) | 23% (0.05) |
| T2D_Remain | 10.9% (0.08) | 10.9% (0.08) | 10.8% (0.08) | 13% (0.08) | 12.4% (0.09) | 9.6% (0.07) | 12.5% (0.09) | 10.9% (0.08) | 9% (0.06) |

*: mean contribution (standard deviation); BP: blood pressure; CVD: cardiovascular diseases; T2D: type 2 diabetes.

**Table G. Relative changes of traits in pleiotropy subgroups.**

| **Phenotype** | **Basic Condition** | **BP** | **CVD** | **Immune system** | **Lipids** | **Obesity** | **Others** | **Respiratory system** | **T2D** |
| --- | --- | --- | --- | --- | --- | --- | --- | --- | --- |
| SBP | -0.013 | 0.009 | 0.01 | -0.025 | -0.015 | -0.015 | 0.026 | -0.019 | 0.047 |
| Hypertension | -0.032 | 0.018 | 0.033 | -0.023 | -0.015 | 0.029 | 0.033 | -0.02 | 0.031 |
| DBP | -0.018 | 0.023 | -0.003 | -0.018 | 0.001 | 0.019 | 0.028 | 0.017 | 0.022 |
| Triglycerides | -0.016 | -0.013 | -0.029 | 0.002 | 0.049 | 0.035 | 0.004 | -0.006 | -0.003 |
| Lipoprotein A | -0.059 | -0.046 | -0.081 | -0.097 | 0.675 | -0.071 | -0.105 | -0.043 | -0.103 |
| LDL direct | 0.002 | -0.059 | -0.057 | -0.047 | 0.183 | -0.021 | -0.004 | -0.042 | -0.022 |
| HDL cholesterol | 0.019 | -0.023 | -0.001 | -0.006 | -0.048 | -0.04 | 0.001 | 0.015 | 0.009 |
| Cholesterol | 0.011 | -0.058 | -0.049 | -0.04 | 0.154 | -0.021 | -0.006 | -0.034 | -0.012 |
| Apolipoprotein B | -0.009 | -0.058 | -0.064 | -0.05 | 0.217 | -0.013 | -0.015 | -0.047 | -0.021 |
| Apolipoprotein A | 0.036 | -0.017 | 0.013 | 0.004 | -0.057 | -0.031 | -0.001 | 0.007 | 0.006 |
| Waist circumference | -0.01 | -0.022 | -0.029 | 0.017 | -0.016 | 0.089 | 0.026 | -0.015 | 0.003 |
| Trunk fat percentage | 0.046 | -0.015 | -0.001 | 0.013 | -0.021 | 0.098 | 0.029 | -0.027 | 0.018 |
| Leg fat percentage | 0.033 | -0.003 | 0.009 | 0.005 | -0.007 | 0.107 | 0.022 | -0.025 | 0.012 |
| Body fat percentage | 0.044 | -0.007 | 0.005 | 0.005 | -0.019 | 0.099 | 0.025 | -0.022 | 0.015 |
| BMI | 0.011 | 0.001 | -0.013 | 0.006 | -0.017 | 0.131 | 0.02 | -0.019 | 0.001 |
| Arm fat percentage | 0.039 | 0.004 | 0.012 | -0.001 | -0.019 | 0.081 | 0.02 | -0.019 | 0.016 |
| Smoking ever | -0.007 | 0.007 | -0.005 | 0.003 | -0.038 | -0.006 | 0.022 | -0.018 | -0.007 |
| Smoking current | -0.017 | 0.014 | 0.004 | -0.022 | -0.035 | 0.002 | 0.003 | 0.002 | 0.019 |
| Smoking | -0.008 | 0.006 | 0.002 | -0.01 | -0.016 | 0.001 | 0.002 | 0.001 | 0.009 |
| Glucose | -0.023 | -0.002 | 0.012 | 0.004 | -0.01 | 0.024 | -0.006 | -0.008 | 0.035 |
| DIA | -0.018 | -0.006 | -0.02 | 0.023 | -0.025 | 0.101 | 0.028 | -0.024 | 0.038 |

BP: blood pressure; CVD: cardiovascular disease; T2D: type 2 diabetes; TDI: Townsend deprivation index; BMI: body mass index; SBP: systolic blood pressure; DBP: diastolic blood pressure; DIA: diabetes.

**Table H. Traits for interaction analysis.**

| FieldID | Trait Name | Group | Data Type | Recode |
| --- | --- | --- | --- | --- |
| 1160 | Sleep duration | Lifestyles | continuous (hrs/day) |  |
| 1200 | Sleeplessness / insomnia | Lifestyles | categorical (3) | Recode into 2 binary variables |
| 1239 | Current tobacco smoking | Lifestyles | categorical (3) | Recode into 2 binary variables |
| 1249 | Past tobacco smoking | Lifestyles | categorical (4) | Recode into 3 binary variables |
| 1269 | Exposure to tobacco smoke at home | Lifestyles | continuous (hrs/week) |  |
| 1279 | Exposure to tobacco smoke outside home | Lifestyles | continuous (hrs/week) |  |
| 1558 | Alcohol intake frequency. | Lifestyles | categorical (6) | Continuous |
| 20116 | Smoking status | Lifestyles | categorical (3) | Recode into 2 binary variables |
| 3062 | Forced vital capacity (FVC) | Physical measures | continuous (litres) |  |
| 3063 | Forced expiratory volume in 1-second (FEV1) | Physical measures | continuous (litres) |  |
| 3064 | Peak expiratory flow (PEF) | Physical measures | continuous (litres/min) |  |
| 4079 | Diastolic blood pressure, automated reading (DBP) | Physical measures | continuous (mmHg) |  |
| 4080 | Systolic blood pressure, automated reading (SBP) | Physical measures | continuous (mmHg) |  |
| 23099 | Body fat percentage | Physical measures | continuous (percent) |  |
| 23111 | Leg fat percentage (right) | Physical measures | continuous (percent) | take the mean |
| 23115 | Leg fat percentage (left) | Physical measures | continuous (percent) |  |
| 23119 | Arm fat percentage (right) | Physical measures | continuous (percent) | take the mean |
| 23123 | Arm fat percentage (left) | Physical measures | continuous (percent) |  |
| 23127 | Trunk fat percentage | Physical measures | continuous (percent) |  |
| 30630 | Apolipoprotein A | Physical measures | continuous (g/L) |  |
| 30640 | Apolipoprotein B | Physical measures | continuous (g/L) |  |
| 30690 | Cholesterol | Physical measures | continuous (mmol/L) |  |
| 30760 | HDL-cholesterol | Physical measures | continuous (mmol/L) |  |
| 30780 | LDL direct | Physical measures | continuous (mmol/L) |  |
| 30790 | Lipoprotein A | Physical measures | continuous (nmol/L) |  |
| 30870 | Triglycerides | Physical measures | continuous (mmol/L) |  |
| 30890 | Vitamin D | Physical measures | continuous (nmol/L) |  |
| 6138 | Qualifications | SES | categorical (7) | Continuous |
| 22189 | Townsend deprivation index at recruitment (TDI) | SES | continuous |  |

**Table I. False Discovery Rate-controlled p-values for interactions between 29 clinical traits and 10 PRSs.**

| **Trait** | **Basic** | **BP** | **CVD** | **Immune** | **Lipids** | **Obesity** | **Others** | **Respir** | **T2D** | **Overall** |
| --- | --- | --- | --- | --- | --- | --- | --- | --- | --- | --- |
| Sleep duration | 9.57E-01 | **1.18E-02** | 9.33E-01 | 8.25E-01 | 2.62E-01 | 9.33E-01 | 8.52E-01 | 8.29E-01 | 6.63E-01 | 1.59E-01 |
| Insomnia (Sometimes) | 7.30E-01 | 3.95E-01 | 6.09E-01 | 5.89E-01 | 9.57E-01 | 9.84E-01 | 3.46E-01 | 7.80E-01 | 9.37E-01 | 2.81E-01 |
| insomnia (Usually) | 3.79E-01 | 8.29E-01 | 6.40E-01 | 7.28E-01 | 4.27E-01 | 8.25E-01 | 8.14E-01 | 8.52E-01 | 7.32E-01 | 9.00E-01 |
| Current tobacco smoking (Most or all days) | 5.00E-01 | 6.85E-01 | 2.97E-01 | **1.91E-03** | 7.34E-02 | 2.73E-01 | **2.65E-02** | **6.08E-06** | 6.77E-02 | **1.47E-13** |
| Current tobacco smoking (Only occasionally) | 3.17E-01 | 6.85E-01 | 6.85E-01 | **6.37E-03** | 2.26E-01 | 8.37E-01 | 4.91E-01 | 2.46E-01 | 1.59E-01 | **4.13E-07** |
| Past tobacco smoking (Most or all days) | 8.24E-01 | 8.64E-01 | 8.41E-01 | 2.46E-01 | 6.63E-01 | 8.14E-01 | 5.87E-01 | 8.14E-01 | 5.09E-01 | 2.94E-01 |
| Past tobacco smoking (Occasionally) | 9.02E-01 | 7.32E-01 | 6.94E-01 | 2.31E-01 | 1.97E-01 | 5.88E-01 | 7.32E-01 | 1.26E-01 | 5.65E-01 | 1.09E-01 |
| Past tobacco smoking (Just tried once or twice) | 8.52E-01 | 7.28E-01 | 5.65E-01 | 9.33E-01 | 8.66E-01 | 9.33E-01 | 4.53E-01 | 8.25E-01 | 9.99E-01 | 6.06E-01 |
| Exposure to tobacco smoke at home | 2.46E-01 | 8.66E-01 | 9.86E-01 | 8.14E-01 | 6.85E-01 | 6.77E-01 | 3.79E-01 | 9.21E-01 | 4.38E-01 | 6.09E-01 |
| Exposure to tobacco smoke outside home | 6.85E-01 | 7.32E-01 | 6.94E-01 | 8.66E-01 | 8.47E-01 | 3.46E-01 | 8.41E-01 | 9.57E-01 | 9.98E-01 | 9.00E-01 |
| Alcohol intake | 3.95E-01 | 6.77E-01 | 9.15E-01 | 7.32E-01 | 6.85E-01 | 7.32E-01 | 3.93E-01 | 4.94E-01 | 9.33E-01 | **2.65E-02** |
| Current smoking | 3.46E-01 | 7.28E-01 | 3.46E-01 | **1.48E-03** | **3.93E-02** | 3.93E-01 | 1.85E-01 | **6.08E-06** | 5.01E-02 | **1.47E-13** |
| Ever smoking | 9.33E-01 | 7.44E-01 | 9.33E-01 | 2.81E-01 | 7.32E-01 | 7.80E-01 | 2.71E-01 | 5.05E-01 | 5.98E-01 | 7.80E-01 |
| FVC | 9.67E-01 | **4.13E-03** | 9.33E-01 | 9.31E-01 | 3.79E-01 | **9.75E-03** | 7.32E-01 | 8.29E-01 | 6.06E-01 | **9.75E-03** |
| FEV1 | 8.52E-01 | **7.32E-03** | 5.65E-01 | 8.52E-01 | 6.77E-01 | **3.01E-02** | 7.19E-01 | 9.43E-01 | 8.24E-01 | **2.11E-02** |
| PEF | 5.50E-01 | 7.34E-02 | 6.94E-01 | 8.52E-01 | 8.57E-01 | 7.34E-02 | 2.28E-01 | 2.26E-01 | 6.98E-01 | **1.20E-02** |
| DBP | 9.00E-01 | 3.93E-01 | 9.00E-01 | 3.82E-01 | 9.15E-01 | 3.79E-01 | 4.46E-01 | 9.84E-01 | 6.97E-01 | 3.95E-01 |
| SBP | 7.62E-01 | 2.58E-01 | 8.79E-01 | 6.63E-01 | 5.88E-01 | 6.94E-01 | 5.01E-02 | 7.48E-01 | 7.98E-01 | 3.99E-01 |
| Body fat percentage | 8.52E-01 | 4.41E-01 | 9.00E-01 | 9.15E-01 | 4.27E-01 | 8.37E-01 | 3.46E-01 | 2.43E-01 | 9.00E-01 | 3.81E-01 |
| Leg fat percentage | 7.32E-01 | 7.32E-01 | 9.85E-01 | 8.14E-01 | 2.31E-01 | 9.14E-01 | 3.95E-01 | 6.06E-01 | 9.86E-01 | 5.65E-01 |
| Arm fat percentage | 9.33E-01 | 5.95E-01 | 9.02E-01 | 9.33E-01 | 9.54E-01 | 9.06E-01 | 9.10E-01 | 1.59E-01 | 9.50E-01 | 9.98E-01 |
| Trunk fat percentage | 9.86E-01 | 2.65E-01 | 9.33E-01 | 6.31E-01 | 5.88E-01 | 7.48E-01 | 2.58E-01 | 2.46E-01 | 8.25E-01 | 1.59E-01 |
| Apolipoprotein A | 9.38E-01 | 9.50E-01 | 8.41E-01 | 7.32E-01 | 8.41E-01 | 8.22E-01 | 9.33E-01 | 9.14E-01 | 3.95E-01 | 7.62E-01 |
| Apolipoprotein B | 6.86E-02 | 8.72E-01 | 8.47E-01 | 4.47E-01 | 7.26E-02 | 7.72E-01 | 7.80E-01 | 8.52E-01 | 5.52E-01 | 3.79E-01 |
| Cholesterol | 2.46E-01 | 8.41E-01 | 7.80E-01 | 7.48E-01 | 6.77E-02 | 6.94E-01 | 9.00E-01 | 9.14E-01 | 3.93E-01 | 6.63E-01 |
| HDL cholesterol | 8.25E-01 | 9.33E-01 | 9.50E-01 | 8.52E-01 | 9.50E-01 | 9.98E-01 | 8.10E-01 | 8.25E-01 | 8.52E-01 | 8.79E-01 |
| LDL direct | 5.01E-02 | 9.34E-01 | 7.32E-01 | 7.32E-01 | **2.99E-02** | 7.32E-01 | 6.85E-01 | 8.41E-01 | 3.95E-01 | 3.38E-01 |
| Lipoprotein A | 7.32E-01 | 9.33E-01 | 6.52E-01 | 9.06E-01 | 8.41E-01 | 9.33E-01 | 9.33E-01 | 4.54E-01 | 8.35E-01 | 2.46E-01 |
| Triglycerides | 8.13E-01 | 9.86E-01 | 9.34E-01 | 8.52E-01 | **2.76E-03** | 7.88E-01 | 8.13E-01 | 7.32E-01 | 3.97E-01 | 3.79E-01 |
| Vitamin D | 3.79E-01 | 9.14E-01 | 9.86E-01 | 7.48E-01 | 3.94E-01 | 3.46E-01 | 1.85E-01 | 3.81E-01 | 9.33E-01 | **9.75E-03** |
| Qualification | 9.47E-01 | 9.34E-01 | 5.98E-01 | 7.80E-01 | 8.63E-01 | 7.32E-01 | 8.47E-01 | 9.00E-01 | 7.62E-01 | 9.02E-01 |
| TDI | 9.06E-01 | 7.32E-01 | 6.85E-01 | 7.28E-01 | 3.17E-01 | 7.28E-01 | 8.13E-01 | 7.80E-01 | 5.05E-01 | 3.79E-01 |

Basic: basic condition; BP: blood pressure; CVD: cardiovascular disease; Immune: immune system; Respir: respiratory system; T2D: type 2 diabetes; FVC: forced vital capacity; FEV1: forced expiratory volume in 1-second; PEF: peak expiratory flow; DBP: diastolic blood pressure; SBP: systolic blood pressure; TDI: Townsend Deprivation Index.

Underlined and bold: < 0.05.

**Table J. False Discovery Rate-controlled p-values for interactions between 29 clinical traits and PRS adjusted for baseline covariates.**

| Trait | Basic | BP | CVD | Immune | Lipids | Obesity | Others | Respir | T2D | overall |
| --- | --- | --- | --- | --- | --- | --- | --- | --- | --- | --- |
| Sleep duration | 9.59E-01 | **1.03E-02** | 8.63E-01 | 9.89E-01 | 2.62E-01 | 8.94E-01 | 7.14E-01 | 8.65E-01 | 4.71E-01 | 1.65E-01 |
| Insomnia (Sometimes) | 5.59E-01 | 4.71E-01 | 6.42E-01 | 4.30E-01 | 8.29E-01 | 9.55E-01 | 4.84E-01 | 8.63E-01 | 8.20E-01 | 4.17E-01 |
| insomnia (Usually) | 2.35E-01 | 9.79E-01 | 4.91E-01 | 5.91E-01 | 2.35E-01 | 6.48E-01 | 9.38E-01 | 9.10E-01 | 8.53E-01 | 4.90E-01 |
| Current tobacco smoking (Most or all days) | 4.96E-01 | 6.42E-01 | 4.03E-01 | **4.23E-03** | 7.70E-02 | 5.14E-01 | 1.16E-01 | **5.53E-05** | 7.10E-02 | **3.11E-09** |
| Current tobacco smoking (Only occasionally) | 2.15E-01 | 5.36E-01 | 4.95E-01 | **7.63E-04** | 1.09E-01 | 7.53E-01 | 2.86E-01 | 1.78E-01 | 1.52E-01 | **3.46E-08** |
| Past tobacco smoking (Most or all days) | 8.94E-01 | 8.92E-01 | 9.89E-01 | 2.35E-01 | 7.55E-01 | 8.70E-01 | 1.72E-01 | 8.92E-01 | 4.95E-01 | 7.11E-01 |
| Past tobacco smoking (Occasionally) | 8.53E-01 | 6.57E-01 | 7.73E-01 | 2.62E-01 | 2.53E-01 | 4.63E-01 | 6.42E-01 | 1.14E-01 | 4.71E-01 | 1.99E-01 |
| Past tobacco smoking (Just tried once or twice) | 6.79E-01 | 4.87E-01 | 6.70E-01 | 9.55E-01 | 7.62E-01 | 9.72E-01 | 2.89E-01 | 8.94E-01 | 8.33E-01 | 7.11E-01 |
| Exposure to tobacco smoke at home | 5.73E-01 | 6.57E-01 | 1.21E-01 | **1.03E-03** | 7.04E-01 | 4.63E-01 | 6.11E-01 | **3.82E-02** | 2.02E-01 | **1.77E-05** |
| Exposure to tobacco smoke outside home | 7.43E-01 | 3.64E-01 | 2.29E-01 | 1.09E-01 | 2.60E-01 | 8.94E-01 | 2.99E-01 | 3.69E-01 | 8.14E-01 | **1.75E-04** |
| Alcohol intake | 5.32E-01 | 6.65E-01 | 9.59E-01 | 4.42E-01 | 5.59E-01 | 6.79E-01 | 3.98E-01 | 3.34E-01 | 9.89E-01 | **3.08E-03** |
| Current smoking | 2.75E-01 | 6.42E-01 | 3.43E-01 | **1.55E-03** | **3.22E-02** | 5.40E-01 | 2.35E-01 | **2.84E-05** | 5.66E-02 | **1.62E-10** |
| Ever smoking | 7.11E-01 | 6.42E-01 | 6.57E-01 | 4.23E-01 | 5.12E-01 | 9.87E-01 | **7.82E-03** | 5.14E-02 | 6.57E-01 | 5.66E-02 |
| FVC | 8.92E-01 | **1.90E-03** | 8.61E-01 | 6.42E-01 | 2.77E-01 | **4.92E-03** | 4.87E-01 | 9.38E-01 | 4.71E-01 | **1.75E-04** |
| FEV1 | 9.59E-01 | **3.08E-03** | 3.47E-01 | 9.89E-01 | 4.30E-01 | **2.72E-02** | 4.91E-01 | 5.57E-01 | 6.79E-01 | **4.36E-04** |
| PEF | 4.96E-01 | 1.04E-01 | 4.75E-01 | 9.11E-01 | 8.26E-01 | 1.09E-01 | 1.42E-01 | 5.66E-02 | 4.79E-01 | **2.82E-03** |
| DBP | 6.57E-01 | **2.04E-02** | 4.17E-01 | 9.55E-01 | 7.14E-01 | 8.62E-01 | 7.62E-01 | 4.95E-01 | 9.67E-01 | 2.16E-01 |
| SBP | 6.06E-01 | **7.82E-03** | 8.76E-01 | 8.29E-01 | 2.86E-01 | 8.60E-01 | 5.70E-01 | 9.53E-01 | 9.40E-01 | 5.57E-01 |
| Body fat percentage | 8.92E-01 | 2.28E-01 | 6.83E-01 | 1.14E-01 | 4.96E-01 | 2.37E-01 | **3.25E-03** | 6.79E-01 | 8.29E-01 | **2.09E-05** |
| Leg fat percentage | 9.89E-01 | 4.12E-01 | 6.42E-01 | 4.12E-01 | 3.61E-01 | 4.71E-01 | **6.48E-03** | 4.71E-01 | 8.92E-01 | **9.39E-05** |
| Arm fat percentage | 8.63E-01 | 2.15E-01 | 4.94E-01 | 1.09E-01 | 8.92E-01 | 1.22E-01 | **1.88E-02** | 6.42E-01 | 8.92E-01 | **5.53E-05** |
| Trunk fat percentage | 7.62E-01 | 1.12E-01 | 6.79E-01 | **3.82E-02** | 5.43E-01 | 2.17E-01 | **2.32E-03** | 8.46E-01 | 7.53E-01 | **5.28E-06** |
| Apolipoprotein A | 9.36E-01 | 9.38E-01 | 9.38E-01 | 4.17E-01 | 9.38E-01 | 4.96E-01 | 8.50E-01 | 8.63E-01 | 4.94E-01 | 4.12E-01 |
| Apolipoprotein B | 1.21E-01 | 6.83E-01 | 9.89E-01 | 6.59E-01 | 6.70E-01 | 5.98E-01 | 2.62E-01 | 9.39E-01 | 2.58E-01 | **1.08E-03** |
| Cholesterol | 2.86E-01 | 8.41E-01 | 9.87E-01 | 7.93E-01 | **9.85E-03** | 7.46E-01 | 8.60E-01 | 8.14E-01 | 3.22E-01 | 8.92E-01 |
| HDL cholesterol | 8.80E-01 | 8.60E-01 | 8.92E-01 | 9.22E-01 | 9.59E-01 | 6.42E-01 | 6.42E-01 | 8.53E-01 | 9.36E-01 | 6.42E-01 |
| LDL direct | 1.05E-01 | 9.22E-01 | 9.36E-01 | 8.61E-01 | **6.49E-04** | 9.82E-01 | 8.92E-01 | 9.22E-01 | 3.93E-01 | 9.91E-01 |
| Lipoprotein A | 8.60E-01 | 9.55E-01 | 8.60E-01 | 9.38E-01 | 2.82E-01 | 9.73E-01 | 8.94E-01 | 2.62E-01 | 7.43E-01 | **4.92E-03** |
| Triglycerides | 9.36E-01 | 9.26E-01 | 5.54E-01 | 8.94E-01 | **3.17E-07** | 2.41E-01 | 7.14E-01 | 1.27E-01 | 5.52E-01 | **2.07E-03** |
| Vitamin D | 2.16E-01 | 8.94E-01 | 9.53E-01 | 5.00E-01 | 2.15E-01 | 1.87E-01 | 8.37E-02 | 1.03E-01 | 8.94E-01 | **4.36E-04** |
| Qualification | 7.93E-01 | 9.89E-01 | 3.98E-01 | 5.59E-01 | 8.62E-01 | 4.96E-01 | 9.36E-01 | 6.79E-01 | 6.42E-01 | 4.96E-01 |
| TDI | 7.73E-01 | 7.11E-01 | 2.84E-01 | 3.61E-01 | 1.16E-01 | 6.79E-01 | 6.79E-01 | 3.22E-01 | 4.17E-01 | **3.82E-02** |

Basic: basic condition; BP: blood pressure; CVD: cardiovascular disease; Immune: immune system; Respir: respiratory system; T2D: type 2 diabetes; FVC: forced vital capacity; FEV1: forced expiratory volume in 1-second; PEF: peak expiratory flow; DBP: diastolic blood pressure; SBP: systolic blood pressure; TDI: Townsend Deprivation Index.

Underlined and bold: < 0.05.

**Table K. False Discovery Rate-controlled p-values for interactions between residuals of 29 clinical traits and PRS.**

| **Trait** | **Basic** | **BP** | **CVD** | **Immune** | **Lipids** | **Obesity** | **Others** | **Respir** | **T2D** | **overall** |
| --- | --- | --- | --- | --- | --- | --- | --- | --- | --- | --- |
| Sleep duration | 9.06E-01 | **3.95E-02** | 9.32E-01 | 9.46E-01 | 5.72E-01 | 9.53E-01 | 9.59E-01 | 8.70E-01 | 8.08E-01 | 6.41E-01 |
| Insomnia (Sometimes) | 9.35E-01 | 5.93E-01 | 9.50E-01 | 8.75E-01 | 6.38E-01 | 9.32E-01 | 8.93E-01 | 9.06E-01 | 8.19E-01 | 8.75E-01 |
| insomnia (Usually) | 7.51E-01 | 8.70E-01 | 7.44E-01 | 9.54E-01 | 4.35E-01 | 8.08E-01 | 8.86E-01 | 9.06E-01 | 7.99E-01 | 1.72E-01 |
| Current tobacco smoking (Most or all days) | 8.75E-01 | 8.75E-01 | 8.70E-01 | 9.53E-01 | 6.17E-01 | 9.73E-01 | 7.94E-01 | **3.63E-03** | 7.51E-01 | 6.42E-01 |
| Current tobacco smoking (Only occasionally) | 8.08E-01 | 8.24E-01 | 9.14E-01 | 8.19E-01 | 8.04E-01 | 8.70E-01 | 8.70E-01 | 7.65E-01 | 8.08E-01 | 4.55E-01 |
| Past tobacco smoking (Most or all days) | 9.83E-01 | 9.06E-01 | 9.83E-01 | 6.79E-01 | 9.32E-01 | 9.51E-01 | 6.42E-01 | 9.51E-01 | 4.17E-01 | 8.59E-01 |
| Past tobacco smoking (Occasionally) | 8.08E-01 | 7.15E-01 | 9.04E-01 | 8.35E-01 | 8.19E-01 | 8.24E-01 | 6.95E-01 | 3.33E-01 | 5.93E-01 | 8.93E-01 |
| Past tobacco smoking (Just tried once or twice) | 8.70E-01 | 9.06E-01 | 8.91E-01 | 7.15E-01 | 7.62E-01 | 8.76E-01 | 7.65E-01 | 9.54E-01 | 8.75E-01 | 3.93E-01 |
| Exposure to tobacco smoke at home | 9.06E-01 | 9.32E-01 | 9.32E-01 | 8.35E-01 | 7.62E-01 | 8.88E-01 | 8.66E-01 | 9.54E-01 | 8.24E-01 | 9.06E-01 |
| Exposure to tobacco smoke outside home | 9.32E-01 | 8.19E-01 | 7.34E-01 | 9.35E-01 | 9.23E-01 | 8.24E-01 | 8.75E-01 | 9.54E-01 | 6.79E-01 | 8.60E-01 |
| Alcohol intake | 6.42E-01 | 7.50E-01 | 9.06E-01 | 8.08E-01 | 7.80E-01 | 8.08E-01 | 4.06E-01 | 6.79E-01 | 9.51E-01 | **2.77E-02** |
| Current smoking | 7.88E-01 | 8.75E-01 | 8.35E-01 | 8.59E-01 | 3.20E-01 | 9.54E-01 | 7.65E-01 | **4.36E-03** | 6.42E-01 | 1.53E-01 |
| Ever smoking | 8.19E-01 | 8.35E-01 | 8.76E-01 | 9.06E-01 | 4.17E-01 | 9.14E-01 | 2.17E-01 | 4.17E-01 | 6.79E-01 | 2.29E-01 |
| FVC | 9.54E-01 | **1.96E-02** | 9.32E-01 | 9.51E-01 | 6.42E-01 | **3.12E-02** | 8.35E-01 | 8.70E-01 | 7.88E-01 | **3.95E-02** |
| FEV1 | 8.86E-01 | **2.73E-02** | 7.26E-01 | 9.06E-01 | 8.08E-01 | 6.88E-02 | 8.08E-01 | 9.54E-01 | 8.60E-01 | **4.33E-02** |
| PEF | 6.79E-01 | 1.89E-01 | 8.70E-01 | 9.06E-01 | 9.54E-01 | 2.29E-01 | 3.93E-01 | 2.60E-01 | 8.08E-01 | 6.88E-02 |
| DBP | 8.88E-01 | 4.86E-01 | 9.06E-01 | 5.72E-01 | 9.53E-01 | 5.72E-01 | 6.42E-01 | 9.51E-01 | 8.19E-01 | 6.60E-01 |
| SBP | 8.08E-01 | 2.29E-01 | 9.32E-01 | 7.20E-01 | 5.93E-01 | 8.08E-01 | 6.88E-02 | 8.70E-01 | 8.93E-01 | 7.04E-01 |
| Body fat percentage | 8.70E-01 | 6.42E-01 | 8.86E-01 | 9.06E-01 | 6.42E-01 | 8.75E-01 | 4.55E-01 | 2.29E-01 | 8.93E-01 | 6.42E-01 |
| Leg fat percentage | 8.59E-01 | 8.70E-01 | 9.18E-01 | 8.70E-01 | 3.93E-01 | 9.23E-01 | 6.79E-01 | 6.92E-01 | 9.51E-01 | 8.08E-01 |
| Arm fat percentage | 8.75E-01 | 7.50E-01 | 8.75E-01 | 9.32E-01 | 9.51E-01 | 9.32E-01 | 9.32E-01 | 1.64E-01 | 9.14E-01 | 9.06E-01 |
| Trunk fat percentage | 9.32E-01 | 3.89E-01 | 9.32E-01 | 7.34E-01 | 7.11E-01 | 8.08E-01 | 2.59E-01 | 2.29E-01 | 8.70E-01 | 2.29E-01 |
| Apolipoprotein A | 9.32E-01 | 9.35E-01 | 8.70E-01 | 8.08E-01 | 8.75E-01 | 8.70E-01 | 9.06E-01 | 9.06E-01 | 5.20E-01 | 8.70E-01 |
| Apolipoprotein B | 1.21E-01 | 8.75E-01 | 9.06E-01 | 6.79E-01 | 9.75E-02 | 8.70E-01 | 8.75E-01 | 9.32E-01 | 7.65E-01 | 6.92E-01 |
| Cholesterol | 3.89E-01 | 8.28E-01 | 8.75E-01 | 8.75E-01 | 6.88E-02 | 8.08E-01 | 9.54E-01 | 9.06E-01 | 6.42E-01 | 8.86E-01 |
| HDL cholesterol | 8.31E-01 | 9.68E-01 | 9.54E-01 | 9.06E-01 | 9.58E-01 | 9.54E-01 | 8.08E-01 | 8.75E-01 | 8.86E-01 | 8.76E-01 |
| LDL direct | 8.24E-02 | 9.06E-01 | 8.60E-01 | 8.72E-01 | **3.12E-02** | 8.59E-01 | 8.19E-01 | 8.93E-01 | 6.79E-01 | 7.16E-01 |
| Lipoprotein A | 6.92E-01 | 7.65E-01 | 8.08E-01 | 8.05E-01 | 9.11E-01 | 9.06E-01 | 8.86E-01 | 6.79E-01 | 9.53E-01 | 6.42E-01 |
| Triglycerides | 9.83E-01 | 9.06E-01 | 9.53E-01 | 9.06E-01 | **3.35E-02** | 8.08E-01 | 7.80E-01 | 8.19E-01 | 8.60E-01 | 3.93E-01 |
| Vitamin D | 6.38E-01 | 9.16E-01 | 9.94E-01 | 8.08E-01 | 6.42E-01 | 6.42E-01 | 2.29E-01 | 5.72E-01 | 9.51E-01 | **2.77E-02** |
| Qualification | 9.14E-01 | 9.53E-01 | 8.08E-01 | 8.08E-01 | 9.06E-01 | 8.08E-01 | 9.54E-01 | 7.65E-01 | 8.88E-01 | 8.14E-01 |
| TDI | 8.35E-01 | 8.08E-01 | 6.79E-01 | 7.94E-01 | 3.93E-01 | 8.08E-01 | 8.08E-01 | 7.94E-01 | 7.65E-01 | 2.48E-01 |

Basic: basic condition; BP: blood pressure; CVD: cardiovascular disease; Immune: immune system; Respir: respiratory system; T2D: type 2 diabetes; FVC: forced vital capacity; FEV1: forced expiratory volume in 1-second; PEF: peak expiratory flow; DBP: diastolic blood pressure; SBP: systolic blood pressure; TDI: Townsend Deprivation Index.

Underlined and bold: < 0.05.

**Table L. Interactions among high-risk subjects.**

| **Subgroup** | **Trait** | **HR (Remain Exposed)** | **HR (Subgroup Unexposed)** | **HR (Subgroup Exposed)** | **P-value for HR** | **RERI** | **P-value for RERI** |
| --- | --- | --- | --- | --- | --- | --- | --- |
| BP | FEV1 | 1.19 (1.03-1.36) | 0.65 (0.58-0.71) | 0.76 (0.63-0.91) | 4.18E-01 | -6.01% | 5.91E-01 |
| BP | FVC | 1.07 (0.9-1.27) | 0.89 (0.8-0.98) | 1.12 (0.96-1.31) | 3.70E-01 | 20.90% | 1.31E-01 |
| BP | Sleep duration | 1.21 (1.08-1.35) | 1.28 (1.09-1.51) | 1.12 (0.75-1.68) | 3.26E-01 | -32.12% | 1.99E-01 |
| Immune | Current smoking | 1.03 (0.93-1.14) | 1.59 (1.39-1.81) | 1.41 (1.1-1.81) | 3.24E-01 | -15.30% | 3.60E-01 |
| Immune | Current smoking (Most or all days) | 1.02 (0.93-1.13) | 1.79 (1.54-2.07) | 1.62 (1.23-2.15) | 4.75E-01 | -10.73% | 5.80E-01 |
| Immune | Current smoking (Only occasionally) | 1.02 (0.93-1.13) | 1.12 (0.87-1.46) | 0.93 (0.55-1.58) | 4.90E-01 | -27.97% | 3.48E-01 |
| Lipids | Current smoking | 1.12 (1.01-1.24) | 1.59 (1.4-1.81) | 1.5 (1.16-1.94) | 2.55E-01 | -7.51% | 6.86E-01 |
| Lipids | LDL | 1.21 (0.99-1.48) | 1.79 (1.18-2.7) | 1.75 (1.15-2.66) | 6.24E-02 | -75.00% | 6.41E-02 |
| Lipids | Triglycerides | 1.23 (0.96-1.57) | 1.58 (1.31-1.9) | 1.57 (1.28-1.92) | 1.32E-01 | -36.97% | 3.30E-01 |
| Obesity | FEV1 | 1.09 (0.91-1.3) | 0.64 (0.58-0.71) | 0.73 (0.59-0.91) | 6.40E-01 | 12.73% | 3.73E-01 |
| Obesity | FVC | 1.12 (0.91-1.38) | 0.91 (0.83-1.01) | 0.99 (0.82-1.2) | 8.58E-01 | 7.67% | 6.65E-01 |
| Others | Current smoking (Most or all days) | 1.01 (0.91-1.12) | 1.69 (1.46-1.97) | 1.94 (1.49-2.53) | 4.09E-01 | 14.27% | 4.79E-01 |
| Respiratory_system | Current smoking | 1.05 (0.93-1.19) | 1.58 (1.39-1.79) | 1.4 (1.05-1.86) | 3.03E-01 | 12.86% | 5.42E-01 |
| Respiratory_system | Current smoking (Most or all days) | 1.05 (0.93-1.19) | 1.78 (1.54-2.06) | 1.61 (1.17-2.21) | 4.23E-01 | 16.04% | 5.06E-01 |

HR: hazard ratio; RERI: relative excess risk due to interaction; FVC: forced vital capacity; FEV1: forced expiratory volume in 1 second; BP: blood pressure.

**Table M. Simulations: interaction tests.**

| Trait | PRS/Subgroup | Beta | Std. Error | t value | Pr(>\|t\|) |
| --- | --- | --- | --- | --- | --- |
| Transformed Trait2 | Trait 2-related PD-PRS | 0.26 | 0.01 | 45.18 | 0.00E+00 |
|  | Trait 3-related PD-PRS | 0.03 | 0.01 | 5.00 | 5.83E-07 |
|  | Trait 4-related PD-PRS | 0.02 | 0.01 | 3.61 | 3.07E-04 |
|  | Others PD-PRS | 0.00 | 0.01 | -0.22 | 8.24E-01 |
|  | Trait 1 PRS | 0.14 | 0.00 | 39.80 | 0.00E+00 |
|  | Trait 2-related Subgroup | 0.49 | 0.06 | 8.60 | 8.56E-18 |
| Transformed Trait3 | Trait 2-related PD-PRS | 0.02 | 0.01 | 3.47 | 5.19E-04 |
|  | Trait 3-related PD-PRS | 0.25 | 0.01 | 46.99 | 0.00E+00 |
|  | Trait 4-related PD-PRS | 0.05 | 0.01 | 9.20 | 3.72E-20 |
|  | Others PD-PRS | 0.01 | 0.01 | 0.95 | 3.41E-01 |
|  | Trait 1 PRS | 0.17 | 0.00 | 50.61 | 0.00E+00 |
|  | Trait 3-related Subgroup | 0.29 | 0.05 | 5.72 | 1.07E-08 |
| Transformed Trait4 | Trait 2-related PD-PRS | 0.03 | 0.01 | 5.20 | 2.01E-07 |
|  | Trait 3-related PD-PRS | 0.05 | 0.01 | 9.97 | 2.08E-23 |
|  | Trait 4-related PD-PRS | 0.25 | 0.01 | 46.49 | 0.00E+00 |
|  | Others PD-PRS | 0.00 | 0.01 | 0.36 | 7.19E-01 |
|  | Trait 1 PRS | 0.18 | 0.00 | 51.53 | 0.00E+00 |
|  | Trait 4-related Subgroup | 0.36 | 0.05 | 7.00 | 2.68E-12 |

**Table N. Simulations: associations between component PRSs defined by Chasman et al. and simulated traits.**

| PRS | Outcome | Beta | Std. Error | t value | Pr(>\|t\|) |
| --- | --- | --- | --- | --- | --- |
| Component 1 PRS | Trait1 | 0.77 | 0.01 | 120.24 | 0.00E+00 |
|  | Trait2 | 0.01 | 0.00 | 3.41 | 6.55E-04 |
|  | Trait3 | 0.00 | 0.00 | -0.87 | 3.87E-01 |
|  | Trait4 | 0.21 | 0.00 | 112.45 | 0.00E+00 |
| Component 2 PRS | Trait1 | 0.66 | 0.01 | 102.66 | 0.00E+00 |
|  | Trait2 | -0.01 | 0.00 | -2.97 | 2.93E-03 |
|  | Trait3 | 0.22 | 0.00 | 116.11 | 0.00E+00 |
|  | Trait4 | -0.01 | 0.00 | -3.93 | 8.57E-05 |
| Component 3 PRS | Trait1 | 0.65 | 0.01 | 99.70 | 0.00E+00 |
|  | Trait2 | 0.19 | 0.00 | 103.18 | 0.00E+00 |
|  | Trait3 | 0.00 | 0.00 | 2.41 | 1.58E-02 |
|  | Trait4 | -0.01 | 0.00 | -3.29 | 1.01E-03 |
| Residual PRS | Trait1 | -0.34 | 0.01 | -52.46 | 0.00E+00 |
|  | Trait2 | -0.03 | 0.00 | -17.27 | 8.13E-67 |
|  | Trait3 | -0.08 | 0.00 | -39.89 | 0.00E+00 |
|  | Trait4 | -0.06 | 0.00 | -29.14 | 2.15E-186 |

**Supplemental Figures**

**Fig A. Genetic correlations between traits.**


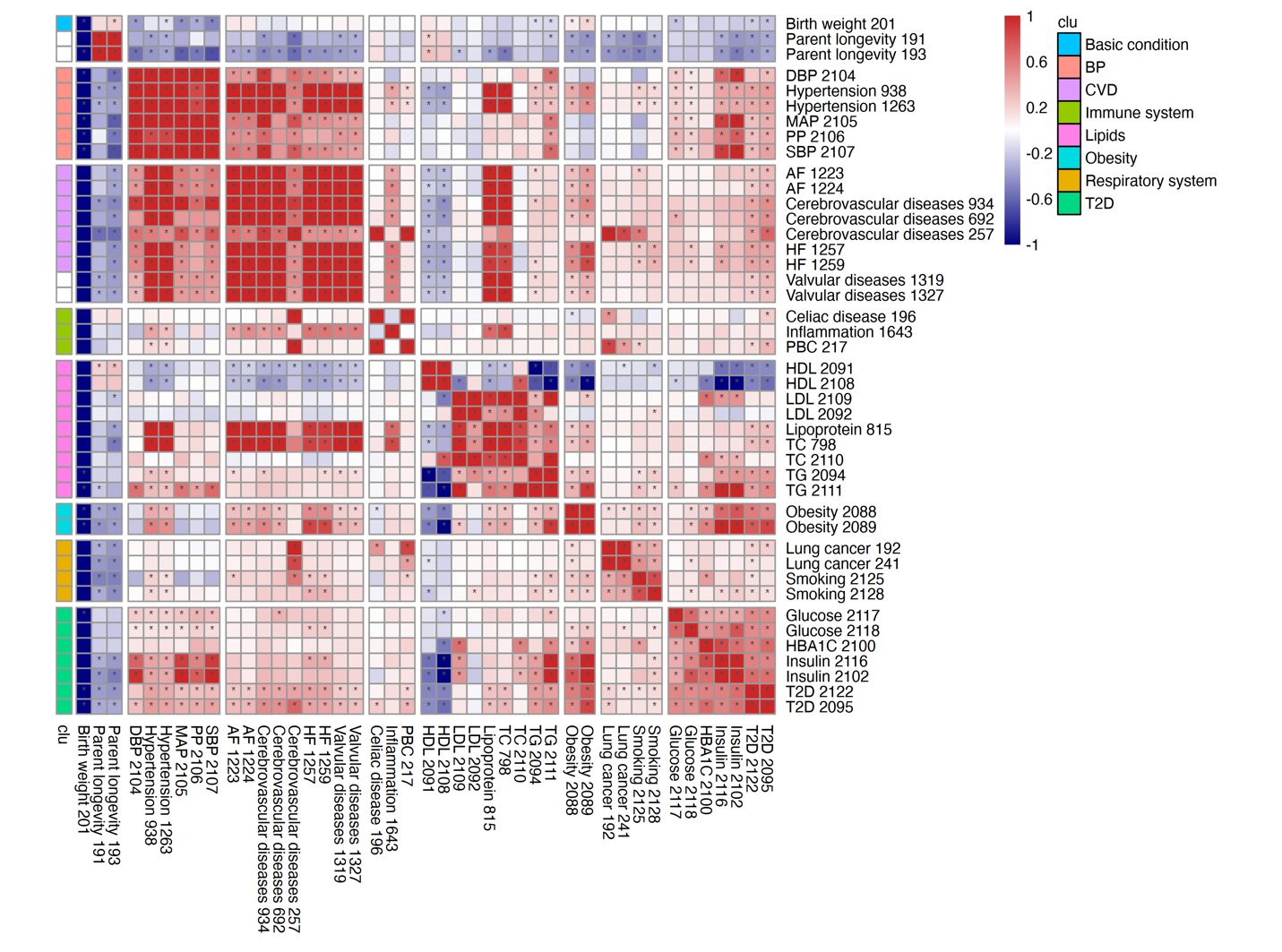


Based on the genetic correlations with CAD, we selected 43 traits and grouped them into 8 clusters based on domestic knowledge. The genetic correlations between 43 selected traits were calculated by GNOVA. The correlation coefficients were shown above and the star indicated a significant genetic correlation after Bonferroni correction (p<0.05/(43*43)). The left bar reflected the pathways we defined.

**Fig B. Hierarchical clustering on genetic correlation matrix.**

**
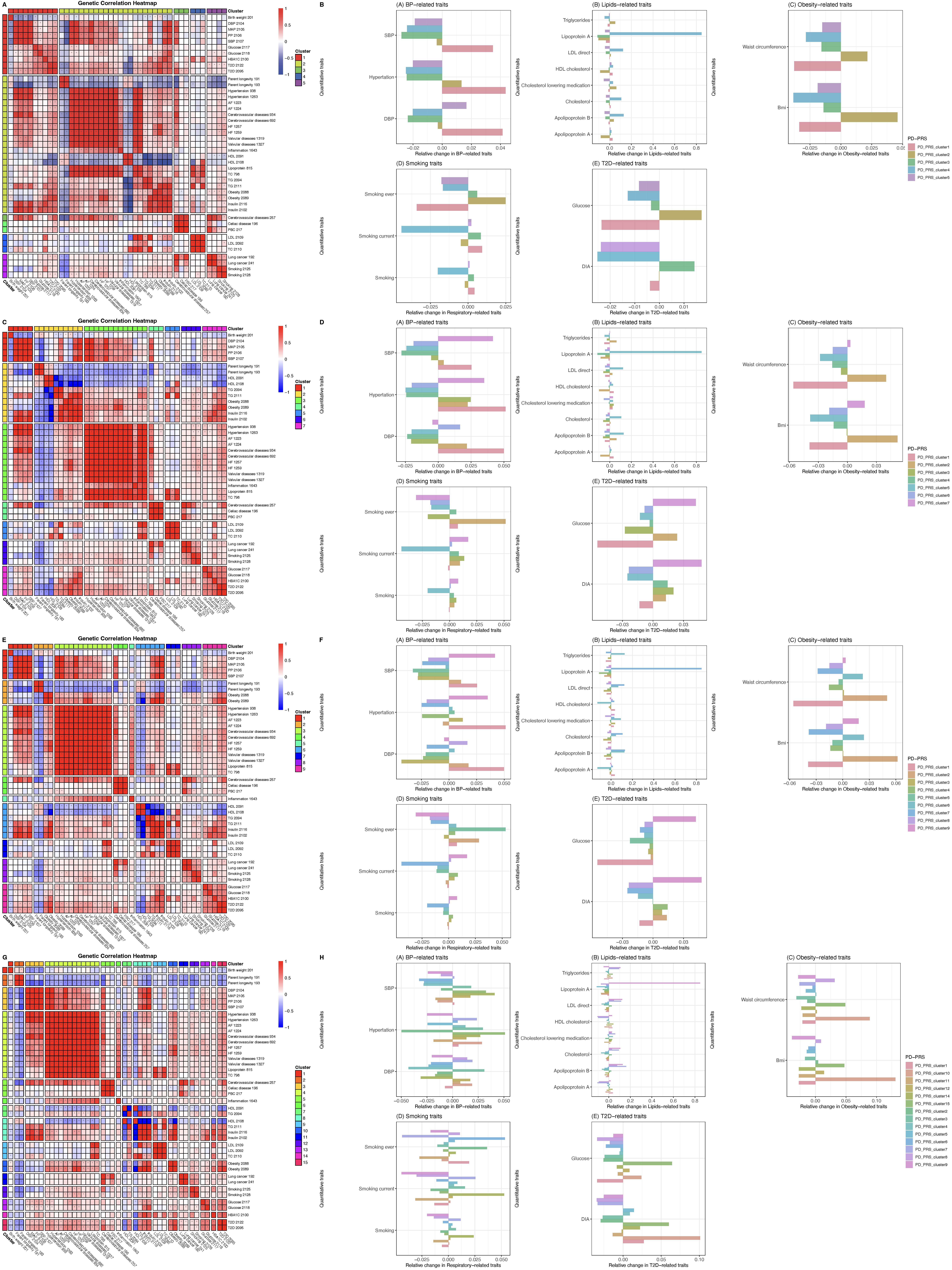
**

Sensitivity analysis of hierarchical clustering was conducted on the genetic correlation matrix derived from 43 selected traits using cluster numbers of 5 (panels A and B), 7 (panels C and D), 9 (panels E and F), and 15 (panels G and H). Panels A, C, E, and G display annotated heatmaps of the genetic correlations, whereas panels B, D, F, and H present the relative changes of the corresponding subgroups. When using 9 clusters (panel C), the resulting clusters were similar to those defined by our domain knowledge, although with reduced interpretability (e.g. parent longevity was grouped with obesity). With fewer clusters (5 and 7), specificity decreased, making it difficult to label the groups and interpret the results; for instance, in the 5-cluster scenario, Cluster 1 was related to both blood pressure and diabetes-related factors. In contrast, increasing the number of clusters to 15 resulted in many clusters with considerable similarity, such as Cluster 13-15 that were all related to diabetes.

Additionally, we divided high-risk subjects into subgroups based on these hierarchical clusters and calculated relative changes to assess phenotypic heterogeneity (Figure S2 B, D, F, H). The phenotypic differences were hard to detect, especially for smoking-related traits.

To align with our goal of generating interpretable PD-PRSs, we decided to define clusters using domain knowledge.

**Fig C. Hazards ratios for CAD of overall CAD PRS, 8 PD-PRSs, and 1 NS-PRS.**
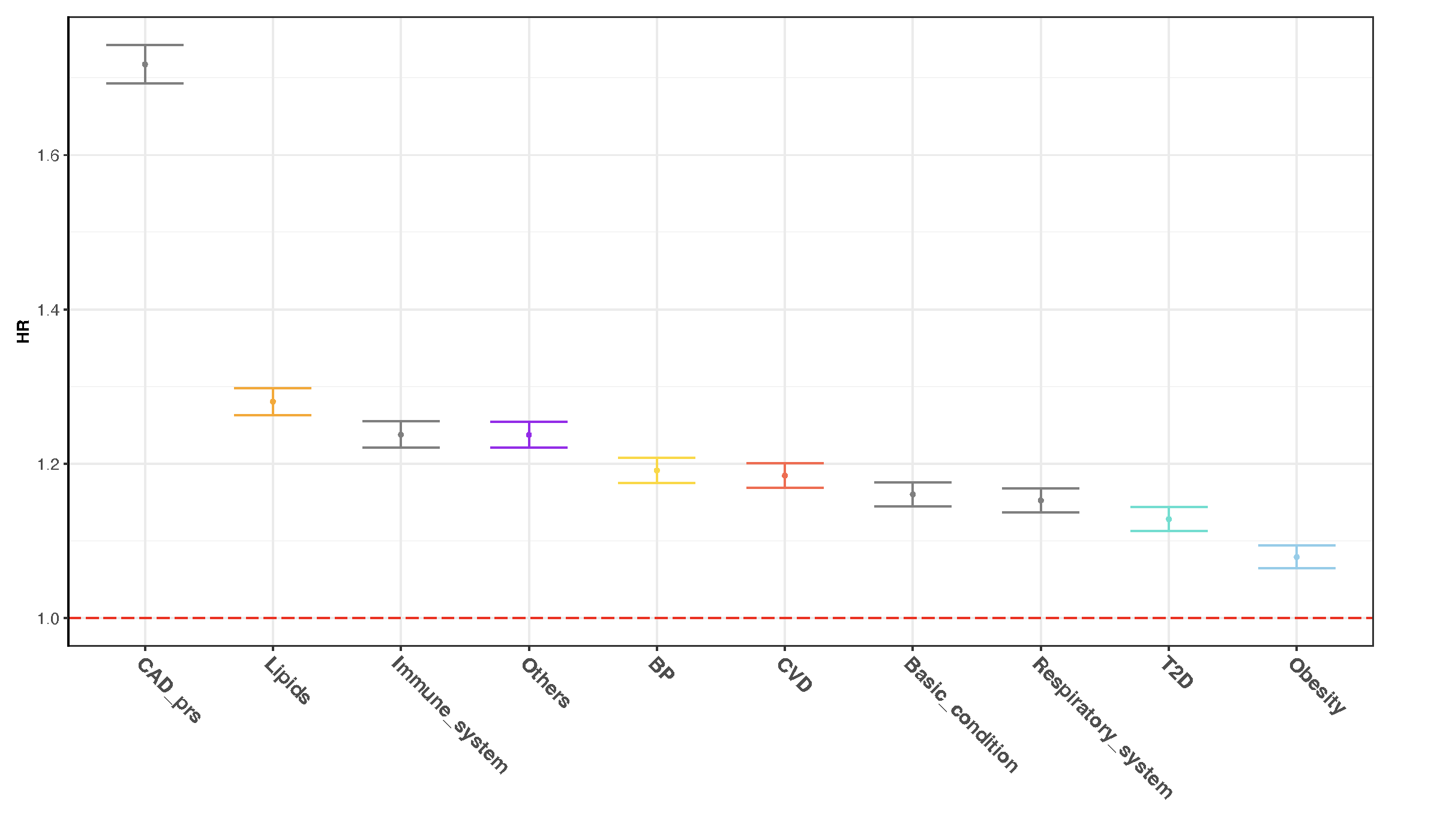
We fitted Cox proportional hazards model for CAD and 10 PRSs (overall CAD PRS and 9 PD-PRSs). The resulted hazards ratios (HRs) were highly correlated with the number of SNPs included in the PRS in a positive way. For example, as the overall CAD PRS included the largest number of SNPs, it showed a much higher HR than other PRSs. Among the PD-PRSs, the Lipids-related PD-PRS had the largest number of SNPs and showed the highest HR.

**Fig D. Number of subjects in 38 subgroup patterns with more than 100 individuals.**

**
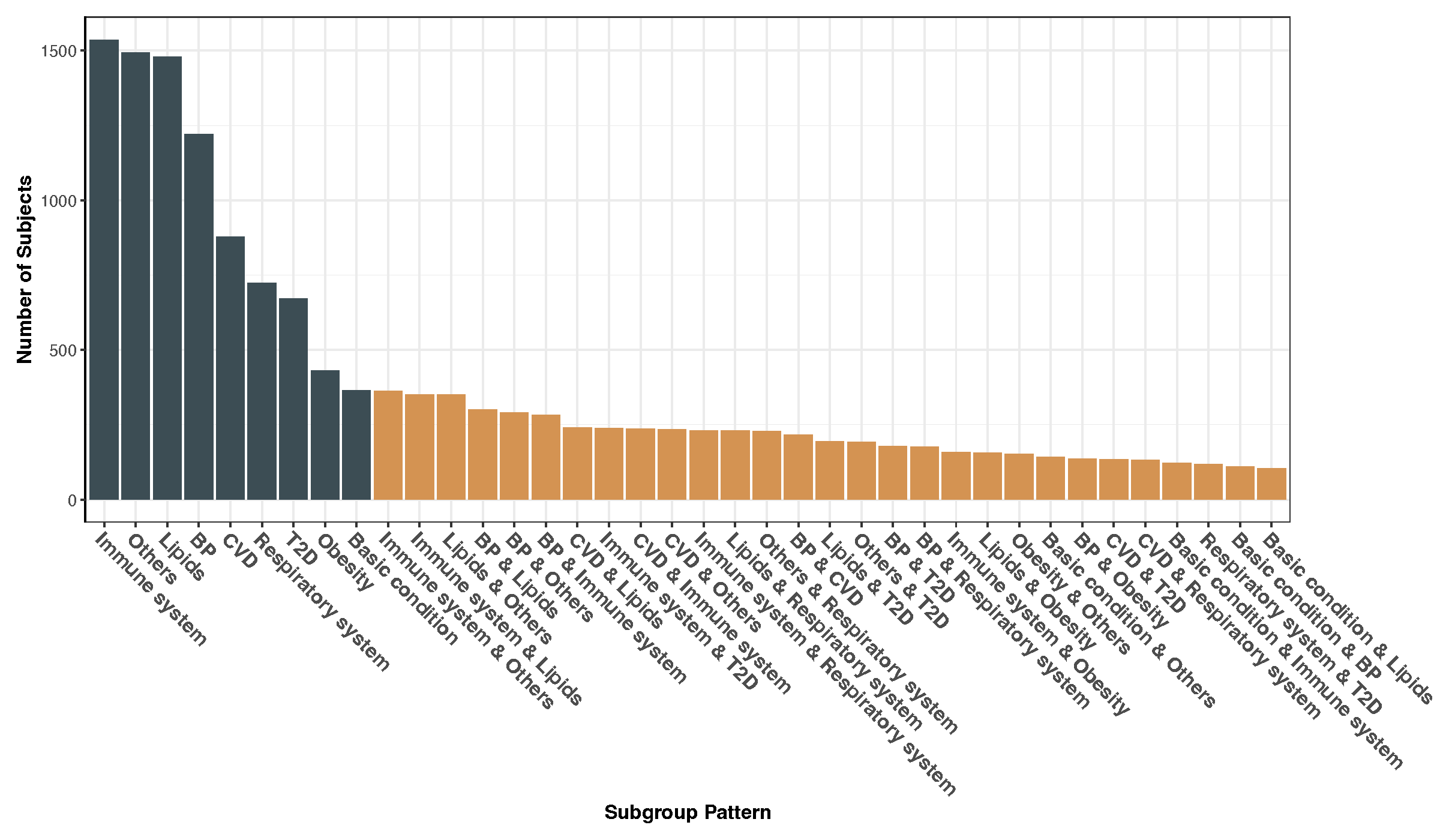
**

We categorized high-risk subjects into 250 subgroup patterns based on the subgroup assignments and only patterns with sample sizes of 100 or more were displayed in the figure. The X-axis represented the subgroup pattern, and the Y-axis quantified the number of subjects in each pattern. Black bars denoted patterns where subjects were assigned to a single subgroup, and yellow bars were the ones with two subgroup assignments. Notably, patterns with single subgroup assignment showed larger number of subjects than other patterns.

**Fig E. Sensitivity analysis for subgroup threshold.**


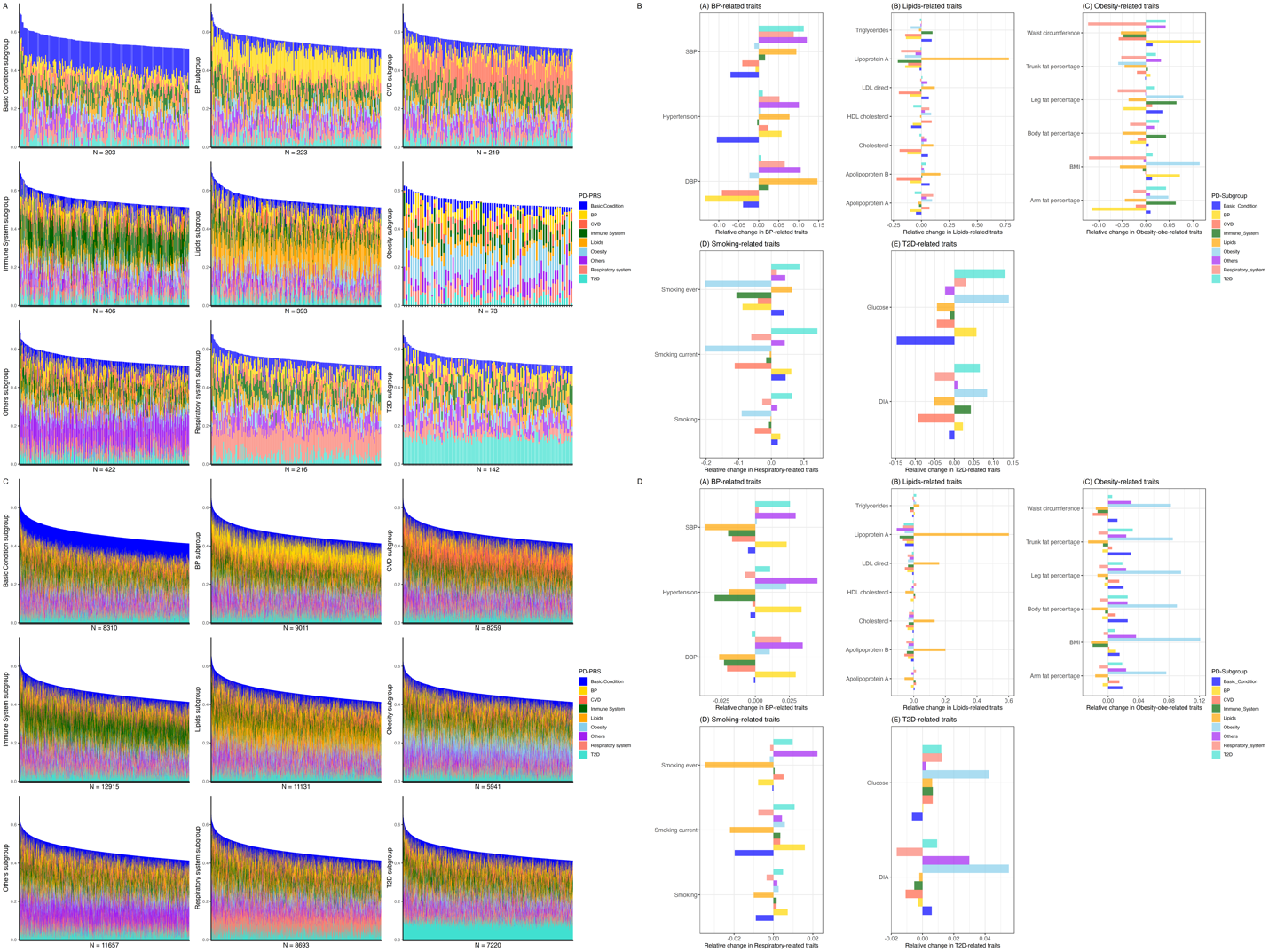


We assessed the robustness of subgroup analysis by defining high-risk subjects with top 1% and 10% PRSs. The PD-PRS profiles were visualized in A for top 1% and B for 10%. The relative differences of traits in subgroups were plotted in C for top 1% and D for 10%.

**Fig F. Interactions between PD-PRS subgroup and cholesterol, LDL, triglycerides, FVC, FEV1, PEF, current smoking and sleep duration.**


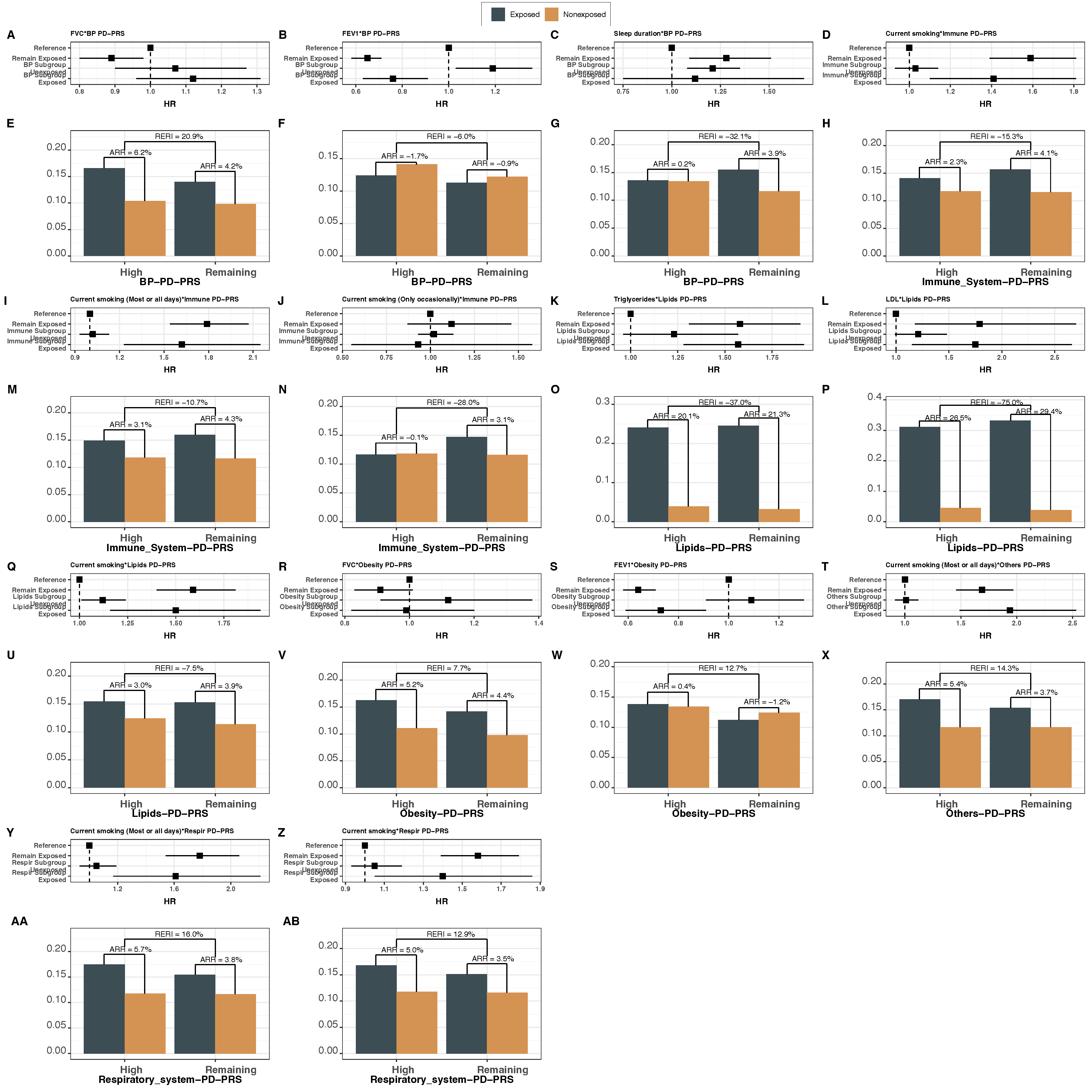


We partitioned the individuals with high CAD PRS into four groups according to their PD-PRS (pathway-specific subgroups versus remaining) and dichotomized traits. The relative risk of three groups compared to the reference group was calculated as HR through Cox proportional hazards model (A-D, I-L, Q-T, Y, Z) The absolute risk in each group was calculated as the incident rate of CAD in the group, and the absolute risk reduction (ARR) reflected the reduction of absolute risk when lowering physical measurements or changing harmful behaviors. The relative excess risk due to interaction (RERI) indicated the proportion of excess risk in the PD-PRS subgroup that could be attributed to the interaction between PD-PRS and corresponding phenotypes (E-H, M-P, U-X, AA, AB)

**Fig G. Simulation results.
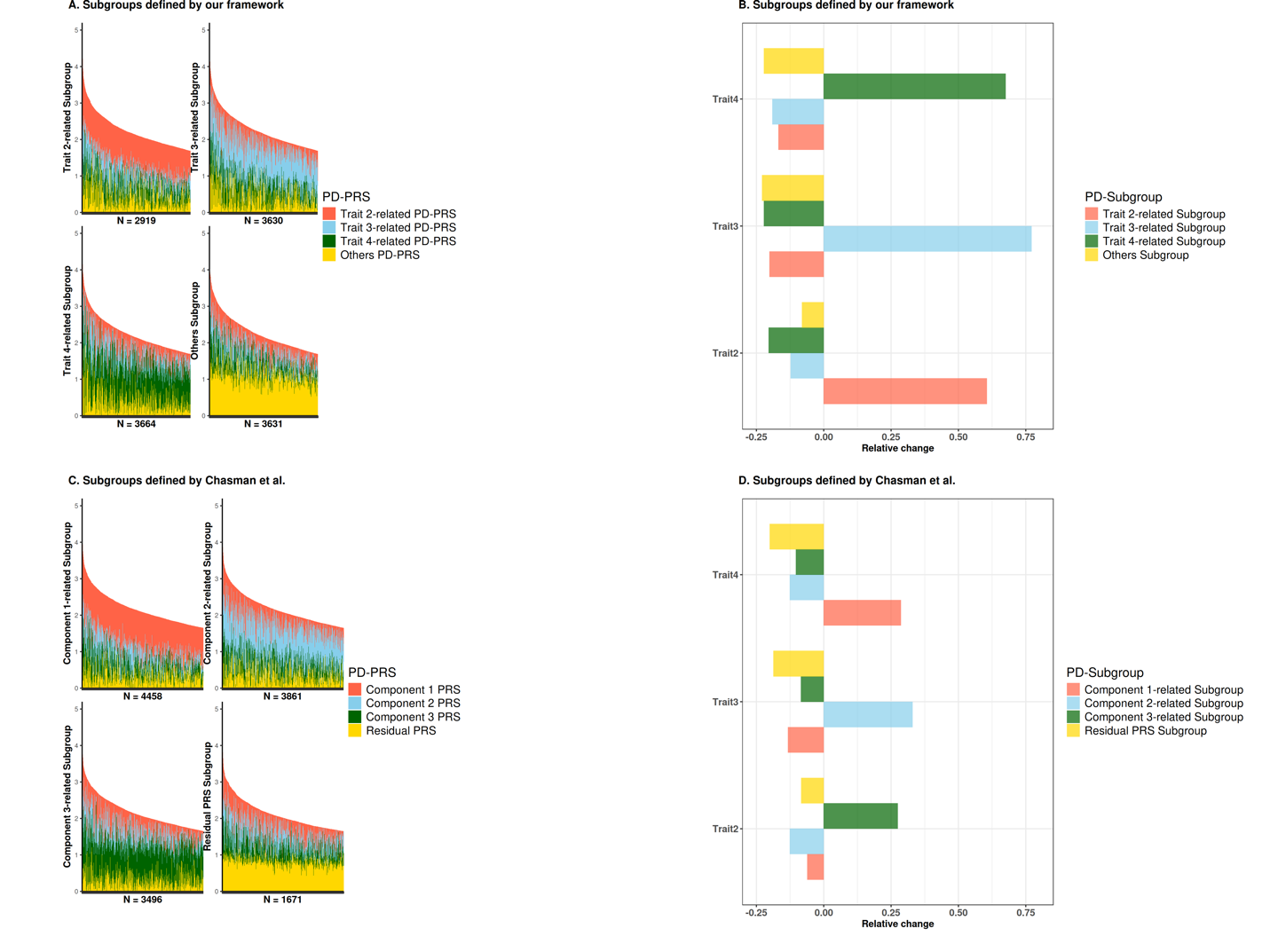
**

To validate the plausibility of our framework, a simulation of GWASs and phenotype for UKB subjects was conducted. With the simulated data, we decomposed the PRS for trait 1 and conducted subgroup analyses using PD-PRS framework and method from Chasman et al. With three PD and one non-specific SNPs subsets, we decomposed the PRS for trait 1 into 4 PD-PRSs: trait 2-related, trait 3-related, trait 4-related, and others (A and B). Using Chasman et al. method, the PRS was decomposed to three component PRSs and one residual PRS (C and D). (A and C) Contributions to the overall PRS. (B and D) The relative change of traits 2-4 comparing subgroups and the corresponding remaining subjects. Both our framework and Chasman et al. can identify the genetic and phenotypic heterogeneity, and our method showed more extreme values in the relative change.

**Fig H. Correlations between 9 PD-PRS.**


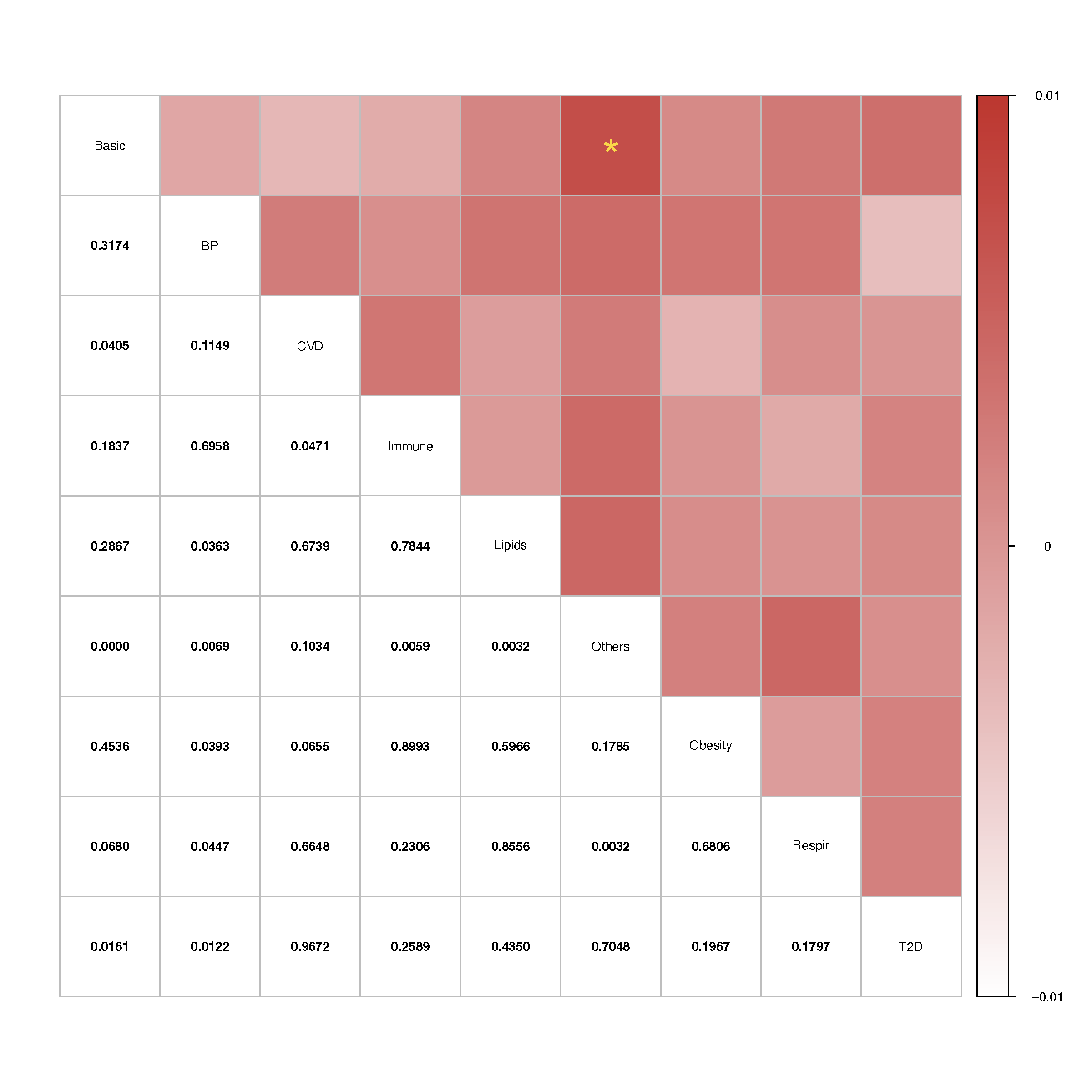


We calculated the correlations between 9 PD-PRSs. The upper right triangle showed the extent of correlation coefficients and the yellow stars indicated significance (p<0.05/(9*9)). The lower left triangle showed the p-values of the correlations. The others PD-PRS was significantly correlated with basic condition PD-PRS, suggesting uncovered functional SNPs within the others PD-PRS.
